# Seven new species of spider-attacking *Hymenoepimecis* Viereck (Hymenoptera, Ichneumonidae, Pimplinae) from Ecuador, French Guiana, and Peru, with an identification key to the world species

**DOI:** 10.3897/zookeys.935.50492

**Published:** 2020-05-21

**Authors:** Diego Galvão de Pádua, Ilari Eerikki Sääksjärvi, Ricardo Ferreira Monteiro, Marcio Luiz de Oliveira

**Affiliations:** 1 Programa de Pós-Graduação em Entomologia, Instituto Nacional de Pesquisas da Amazônia, Av. André Araújo, 2936, Petrópolis, 69067-375, Manaus, Amazonas, Brazil Instituto Nacional de Pesquisas da Amazônia Manaus Brazil; 2 Biodiversity Unit, Zoological Museum, University of Turku, FIN-20014, Turku, Finland University of Turku Turku Finland; 3 Laboratório de Ecologia de Insetos, Depto. de Ecologia, Universidade Federal do Rio de Janeiro, Av. Carlos Chagas Filho, 373, Cidade Universitária, Ilha do Fundão, 21941-971, Rio de Janeiro, Rio de Janeiro, Brazil Universidade Federal do Rio de Janeiro Rio de Janeiro Brazil

**Keywords:** Amazonia, Andes, biodiversity, koinobiont, Neotropical, *Polysphincta* genus group, parasitoids, rain forest

## Abstract

Seven new species of *Hymenoepimecis* Viereck are described from Peruvian Andes and Amazonia, French Guiana and Ecuador: *H. andina* Pádua & Sääksjärvi, **sp. nov.**, *H. castilloi* Pádua & Sääksjärvi, **sp. nov.**, *H. dolichocarinata* Pádua & Sääksjärvi, **sp. nov.**, *H. ecuatoriana* Pádua & Sääksjärvi, **sp. nov.**, *H. longilobus* Pádua & Sääksjärvi, **sp. nov.**, *H. pucallpina* Pádua & Sääksjärvi, **sp. nov.**, and *H. rafaelmartinezi* Pádua & Sääksjärvi, **sp. nov.** In addition, the male of the *Hymenoepimecis kleini* Pádua & Sobczak, 2015 is described, new faunistic records for the genus provided, as well as an illustrated identification key to all known species of the genus.

## Introduction

*Hymenoepimecis* Viereck, 1912 is a moderately large genus of spider-attacking Darwin wasps. It is confined to the New World where it occurs from Mexico and Central America to Southern Brazil (Gauld 1991; Kumagai and Graf 2002; Yu et al. 2012; Pádua et al. 2015).

The species of *Hymenoepimecis* are known to be koinobiont ectoparasitoids attacking sub-adult and adult orb-web-spinning spiders belonging to the families Araneidae and Tetragnathidae (summarised in Pádua et al. 2016). *Hymenoepimecis* is closely related to *Acrotaphus* Townes and *Ticapimpla* Gauld and it can be easily separated from these two genera by possessing a pocket-like structure on the mediodorsal part of the pronotum (Gauld 1991).

Currently, there are 20 described species in the genus (Yu et al. 2012; Pádua et al. 2015) and the majority of them have been discovered from Brazil (Brullé 1846; Kriechbaumer 1890; Loffredo and Penteado-Dias 2009; Sobczak et al. 2009; Pádua et al. 2015) and Central America (see Cresson 1865; Brues and Richardson 1913; Townes and Townes 1966; Gauld 1991, 2000). Very little is known about the occurrence of the genus in other Latin American countries. The main aim of this work is to describe seven new species of *Hymenoepimecis* from Peru, French Guiana and Ecuador. In addition, we provide new faunistic records for the genus and an illustrated identification key to all species.

## Materials and methods

The specimens studied in this work are deposited at the Florida State Collection of Arthropods (**FSCA**), Gainesville, USA, The Natural History Museum, University of San Marcos (**MUSM**), Lima, Peru, The Natural History Museum, London, UK (**NHM**), and the Biodiversity Unit, Zoological Museum, University of Turku (**ZMUT**), Turku, Finland. The specimens of MUSM are currently on loan in ZMUT.

Specimens were examined with an Olympus SZ61 and SZX10 stereomicroscopes and measurements were made through a millimetre ocular. Digital layer images were taken using a Canon DS126461 digital camera attached to an Olympus SZX16 stereomicroscope and combined by using the software Zerene Stacker (Version 1.04 Build T201706041920). Drawings were vectorized digitally using Adobe Illustrator.

The morphological terminology and style of descriptions follow those of Pádua et al. (2015). In this study, the measures and proportions between the structures are given as the value of the holotype [in brackets], followed by the minimum and maximum number of variations. The new country records of the genus are marked with a double asterisk and new country records of the species were marked with a single asterisk. The maps were produced through the website Simplemappr (https://www.simplemappr.net).

### Key to the world species of Hymenoepimecis

[The female of the *H. cameroni* and males of *H. amazonensis*, *H. atriceps*, *H. castilloi* sp. nov., *H. heidyae*, *H. heteropus*, *H. jordanensis*, *H. neotropica*, *H. pucallpina* sp. nov., *H. silvanae*, and *H. sooretama* are unknown].

**Table:** 

1	Female; ovipositor projecting conspicuously beyond the apex of metasoma	**2**
–	Male	**27**
2	Face sculptured below the insertion of antennae, with a longitudinal carina in the middle (Figs 7, 12, 15); head with occipital carina projected, not curved upwards, with a dorsal concavity in the apex (Figs 22, 27, 30); pronotum with a pocket-like structure reduced longitudinally (Figs 22, 27, 30); sternite I with a spine-like ventral projection posteriorly (Figs 52, 57, 60)	**(*H. jordanensis* species group) 3**
–	Face not sculptured below the insertion of antennae, without a longitudinal carina in the middle (Figs 1–6, 8–11, 13, 14); head with occipital carina projected and curved upwards, without a dorsal concavity in the apex (Figs 16–21, 23–26, 28, 29); pronotum with the pocket-like structure not reduced longitudinally (Figs 31–36, 38–41, 43, 44); sternite I with a low, rounded swelling posteriorly (Figs 31–36, 38–41, 43, 44) or with a high, laterally compressed, nasute ventral protuberance (Fig. 47)	**7**
3	Wing bicoloured (blackish, with one yellowish hyaline band) (Fig. 75) or fore wing yellowish, with two blackish bands (Fig. 72)	**4**
–	Wing monocoloured (hyaline, without band(s)) (Fig. 67)	**5**
4	Fore wing blackish, with a yellowish hyaline preapical band; hind leg black, with base of coxa orange (Fig. 75)	***H. uberensis* Pádua & Onody, 2015**
–	Fore wing hyaline yellowish, with apex blackish and with a blackish preapical band; hind leg orange with femur, tibia and tarsus blackish brown (Fig. 72)	***H. rafaelmartinezi* sp. nov.**
5	Metasoma darkish brown, with whitish lateral marks on anterior margins of tergites II–V (e.g., Fig. 61)	***H. jordanensis* Loffredo & Penteado-Dias, 2009**
–	Metasoma orange, with blackish marks on posterior margins in some tergites (Fig. 67)	**6**
6	Tarsal claw with a flat preapical tooth, apex of claw 3.0 times the length of the tooth (Fig. 82)	***H. kleini* Pádua & Sobczak, 2015**
–	Tarsal claw with a short basal lobe vertically, more or less square, apex of claw clearly overtaking the lobe (Fig. 95)	***H. amazonensis* Pádua & Oliveira, 2015**
7	Epicnemial carina present (Figs 34, 44, 96A)	**8**
–	Epicnemial carina absent (Figs 31–33, 35–43, 45)	**10**
8	Fore wing black (Fig. 92); submetapleural carina present (Fig. 96B)	***H. argyraphaga* Gauld, 2000**
–	Fore wing hyaline (Figs 61, 63, 64, 69, 74); submetapleural carina absent	**9**
9	Mesosoma entirely orange (Fig. 34); metasoma orange, with posterior margins of tergites II–IV narrowly black, tergites V+ black (Fig. 64)	***H. dolichocarinata* sp. nov.**
–	Mesosoma orange, with propleuron, pronotum, dorsal half of metapleuron and propodeum blackish; metasoma entirely blackish (Fig. 74)	***H. tedfordi* Gauld, 1991**
10	Sternite with a high, nasute ventral protuberance (Fig. 47)	**11**
–	Sternite I with a low, rounded swelling posteriorly (Figs 46, 48–51, 53–56, 58, 59)	**13**
11	Fore wing yellowish hyaline, with black apex (Fig. 62)	***H. bicolor* (Brullé, 1846)**
–	Fore wing hyaline (sometimes with apex slightly blackish) (Figs 61, 63, 64, 69, 74)	**12**
12	Hind leg black, except base of coxa orange	***H. heidyae* Gauld, 1991**
–	Hind leg orange, with distal 0.5 of tibia and tarsus blackish (Fig. 93)	***H. robertsae* Gauld, 1991**
13	Wing monocoloured (hyaline or yellowish hyaline or blackish) (Figs 61, 63, 64, 69, 74, 91, 92)	**14**
–	Wing bicoloured (with one band or two bands) (Figs 65, 66, 68, 70, 71)	**20**
14	Metasoma reddish brown	**15**
–	Metasoma mainly darkish brown to black or orange (Figs 61–75)	**16**
15	Fore wing hyaline (e.g., Fig. 92)	***H. atriceps* (Cresson, 1865)**
–	Fore wing blackish, with pterostigma yellow (Fig. 91)	***H. veranii* Loffredo & Penteado-Dias, 2009**
16	Metasoma entirely black, tergites without whitish anterior margin (Fig. 63)	***H. castilloi* sp. nov.**
–	Metasoma blackish or darkish brown, some tergites with whitish anterior margin (e.g., Fig. 61) or metasoma orange, some tergites with posterior margin blackish (Fig. 69)	**17**
17	Metasoma blackish or blackish brown, some tergites with anterior margin whitish (e.g., Figs 61)	**18**
–	Metasoma orange, some tergites with posterior margin blackish (Fig. 69)	**19**
18	Occipital carina only slightly projected (Figs 31, 33); ovipositor < 1.2 times as long as hind tibia	***H. andina* sp. nov.**
–	Occipital carina clearly projected (e.g., Figs 32, 38); ovipositor > 1.4 times as long as hind tibia	***H. sooretama*Sobczak et al., 2009**
19	Posterior ocelli separated from eyes by 0.4 times its own maximum diameter, in dorsal view; fore wing fuscous (see Sobczak et al. 2009); ovipositor 0.9 times as long as hind tibia; hind leg brownish, except base of coxa orange	***H. japi*Sobczak et al., 2009**
–	Posterior ocelli separated from eyes by 0.6–0.8 times its own maximum diameter, in dorsal view; fore wing hyaline; ovipositor 1.1–1.3 times as long as hind tibia; hind leg orange, with femur, tibia and tarsus blackish (Figs 69)	***H. manauara* Pádua & Oliveira, 2015**
20	Fore wing blackish, with a yellowish band more or less in median region (Fig. 71)	**21**
–	Fore wing yellowish hyaline, with two black bands (Figs 65, 66, 68)	**22**
21	Occipital carina reduced in dorsal part (Fig. 43); metasoma orange, with tergites VI+ black (Fig. 73)	***H. ribeiroi* Pádua & Sobczak, 2015**
–	Occipital carina not reduced in dorsal part (Fig. 41); metasoma entirely black (Fig. 71)	***H. pucallpina* sp. nov.**
22	Tarsal claw with a preapical tooth (Fig. 85)	***H. neotropica* (Brues & Richardson, 1913)**
–	Tarsal claw with a longitudinally elongated lobe or with a square lobe (Figs 76–81, 83, 84, 86–90)	**23**
23	Tarsal claw with a longitudinally elongated lobe (Fig. 83)	***H. longilobus* sp. nov.**
–	Tarsal claw with a more or less square lobe (Figs 76–81, 84, 86–90)	**24**
24	Metasoma black, with tergite I orange (Fig. 94)	***H. heteropus* (Kriechbaumer, 1890)**
–	Metasoma entirely brownish orange or orange, with posterior tergites blackish (Figs 65, 66, 68, 70)	**25**
25	Metasoma brownish orange or ferruginous (see Loffredo & Penteado-Dias 2009)	***H. silvanae* Loffredo & Penteado-Dias, 2009**
–	Metasoma orange, with posterior tergites blackish (Figs 65, 66, 68, 70)	**26**
26	Ovipositor > 1.5 times as long as hind tibia; pronotum black (in general) (Fig. 35)	***H. duckensis* Pádua & Onody, 2015**
–	Ovipositor < 1.3 times as long as hind tibia; pronotum orange (Fig. 36)	***H. ecuatoriana* sp. nov.**
27	Face sculptured below the insertion of antennae, with a longitudinal carina in the middle part (Figs 7, 12, 15); head with occipital carina projected, not curved upwards, with a concavity dorsally in the apex (Figs 22, 27, 30); pronotum with the pocket-like structure reduced longitudinally (Figs 22, 27, 30); sternite I with a spine-like ventral projection posteriorly (Figs 52, 57, 60)	**(*H. jordanensis* species group) 28**
–	Face not sculptured below the insertion of antennae, without a longitudinal carina in the middle part (Figs 1–6, 8–11, 13, 14); head with occipital carina projected and curved upwards, without a concavity in the apex dorsal (Figs 16–21, 23–26, 28, 29); pronotum with the pocket-like structure not reduced longitudinally (Figs 31–36, 38–41, 43, 44); sternite I with a low, rounded swelling posteriorly (Figs 31–36, 38–41, 43, 44) or with a high, laterally compressed, nasute ventral protuberance (Fig. 47)	**30**
28	Fore wing hyaline (Fig. 103)	***H. kleini* Pádua & Sobczak, 2015**
–	Fore wing blackish, with one yellowish hyaline band (Fig. 106) or fore wing yellowish, with two blackish bands (Figs 100, 102, 104)	**29**
29	Fore wing blackish, with yellowish hyaline band between junction of vein R1 up to pterostigma until half vein M; hind leg black, with base of coxa orange (Fig. 106)	***H. uberensis* Pádua & Onody, 2015**
–	Fore wing hyaline yellowish, with apex distal to 2rs-m blackish, and with a blackish median band extending backwards from anterior margin, to veins Rs+M and junction of pterostigma with vein R1; hind leg orange with femur, tibia and tarsus blackish brown (Fig. 104)	***H. rafaelmartinezi* sp. nov.**
30	Epicnemial carina present (Figs 34, 44, 96)	**1**
–	Epicnemial carina absent (Figs 31–33, 35–43, 45)	**34**
31	Fore wing hyaline (Figs 97, 99, 101, 103)	**32**
–	Fore wing blackish (Fig. 92)	**33**
32	Mesosoma entirely orange; metasoma orange, with posterior margins of tergites II–IV narrowly black, tergites V+ black (Fig. 99); margin of the gena flat behind the eyes (Fig. 19)	***H. dolichocarinata* sp. nov.**
–	Mesosoma orange, with pronotum, metapleuron and propodeum blackish; metasoma entirely black (Fig. 74); margin of gena strongly narrowed behind eyes (see female in Fig. 29)	***H. tedfordi* Gauld, 1991**
33	Submetapleural carina present (Fig. 96B)	***H. argyraphaga* Gauld, 2000**
–	Submetapleural carina absent	***H. cameroni* Townes, 1966**
34	Sternite I with a high, laterally compressed ventral protuberance (Fig. 47)	**35**
–	Sternite I with a low, rounded swelling posteriorly (Figs 46, 48–51, 53–56, 58, 59)	**36**
35	Fore wing yellowish hyaline, with black apex; hind leg orange, with femur, tibia and tarsus black (Fig. 98)	***H. bicolor* (Brullé, 1846)**
–	Fore wing hyaline (sometimes with apex slightly blackish) (e.g., Figs 99, 101); hind leg orange, with 0.5 distal of tibia and tarsus blackish (Fig. 93)	***H. robertsae* Gauld, 1991**
36	Wing bicoloured (with one band or two bands) (Figs 100, 102, 104)	**37**
–	Wing monocoloured (hyaline or yellowish hyaline or fuscous or blackish) (Figs 91, 97, 99)	**39**
37	Fore wing blackish, with one yellowish band in median region (sometimes fore wing with base yellowish) (Fig. 105); occipital carina reduced in dorsal part (Fig. 43)	***H. ribeiroi* Pádua & Sobczak, 2015**
–	Fore wing yellowish, with two black bands (Figs 100, 102, 104); occipital carina not reduced in dorsal part (Figs 35, 38, 40); metasoma blackish (Fig. 97)	**38**
38	Pronotum entirely black or orange, with anterior region black (Fig. 100)	***H. duckensis* Pádua & Onody, 2015**
–	Pronotum entirely orange (the Peruvian specimens have anterior margin of pronotum blackish) (Fig. 102)	***H. longilobus* sp. nov.**
39	Fore wing blackish, with pterostigma yellow (Fig. 91)	***H. veranii* Loffredo & Penteado-Dias, 2009**
–	Fore wing hyaline (Figs 99, 101, 103)	**40**
40	Occipital carina only slightly projected and not curved upwards dorsally (Figs 16, 31); metasoma darkish brown, with whitish lateral marks on anterior margins of tergites II–V (Fig. 97)	***H. andina* sp. nov.**
–	Occipital carina clearly projected and curved upwards dorsally (Figs 24, 39); metasoma orange, with some tergites with posterior margins black (Fig. 103)	**41**
41	Posterior ocelli separated from eyes by more or less 0.4 times its own maximum diameter, in dorsal view; fore wing fuscous (see Sobczak et al. 2009); hind leg brownish, except base of coxa orange	***H. japi*Sobczak et al., 2009**
–	Posterior ocelli separated from eyes by 0.6–0.8 times its own maximum diameter, in dorsal view; fore wing hyaline (Fig. 24); hind leg orange, with femur, tibia and tarsus blackish (there are specimens with femur basal half orange and apical half blackish or femur orange with apex blackish) (Fig. 103)	***H. manauara* Pádua & Oliveira, 2015**

## Faunistics and taxonomy

### Hymenoepimecis

Taxon classificationAnimaliaHymenopteraIchneumonidae

Viereck, 1912

2B775A64-9B5E-543B-BCEC-11187EE95D39

Epimecis Brullé, 1846: 112. Type-species: Epimecis bicolor Brullé, by subsequent designation; Ashmead, 1900: 54.
Hymenoepimecis Viereck, 1912: 149. [Replacement name for Epimecis Brullé, 1846, junior homonym of Epimecis Hübner, 1825]

#### Comments.

According to Gauld (1991, 2000) *Hymenoepimecis* is very similar with the sister genus *Acrotaphus* Townes, 1960. They both have the occipital carina strongly raised (flange-like) and projecting posteriorly to surround the anterior reflexed end of the pronotum; head rounded with the genae strongly narrowed from the eyes to the occipital flange; and the pronotum unusually elongated, with a long horizontal part mediodorsally. It differs from *Acrotaphus* by having a unique forwardly directed pocket-like structure on the pronotum mediodorsally.

### Hymenoepimecis andina

Taxon classificationAnimaliaHymenopteraIchneumonidae

Pádua & Sääksjärvi sp. nov.

F8BC4922-EDB0-5EA3-8184-021049397F9F

http://zoobank.org/98DE0A8A-6E2D-4B2C-BD1D-1C0416E067D1

**Figures 1–15.**
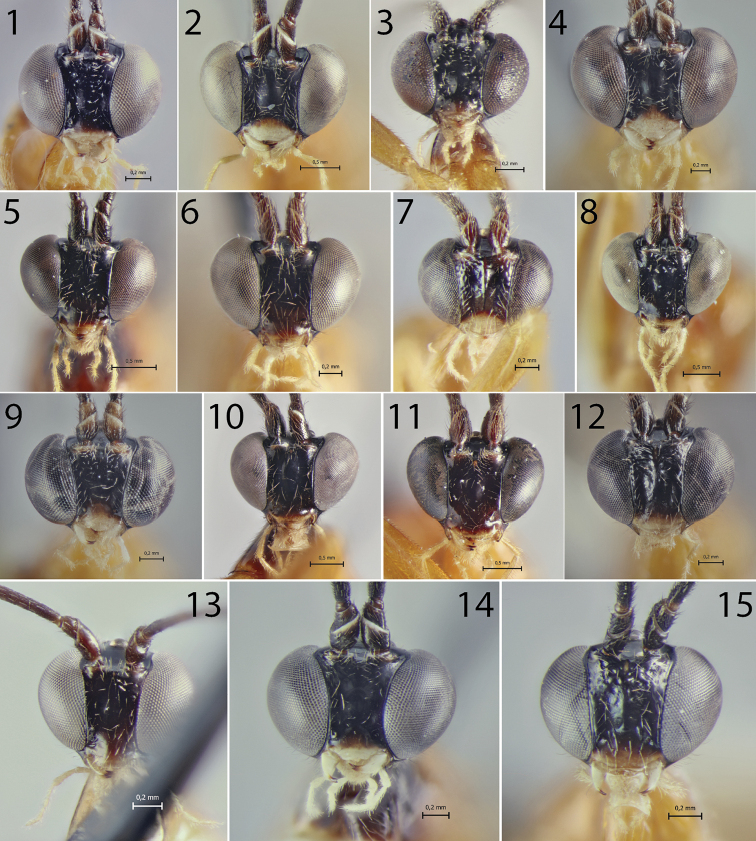
Heads of *Hymenoepimecis* spp. (females), frontal view **1***H. andina* sp. nov. (holotype) **2***H. bicolor***3***H. castilloi* sp. nov. (holotype) **4***H. dolichocarinata* sp. nov. (holotype) **5***H. duckensis***6***H. ecuatoriana* sp. nov. (holotype) **7***H. kleini***8***H. longilobus* sp. nov. (holotype) **9***H. manauara***10***H. neotropica***11***H. pucallpina* sp. nov. (holotype) **12***H. rafaelmartinezi* sp. nov. (holotype) **13***H. ribeiroi***14***H. tedfordi***15***H. uberensis*.

**Figures 16–30.**
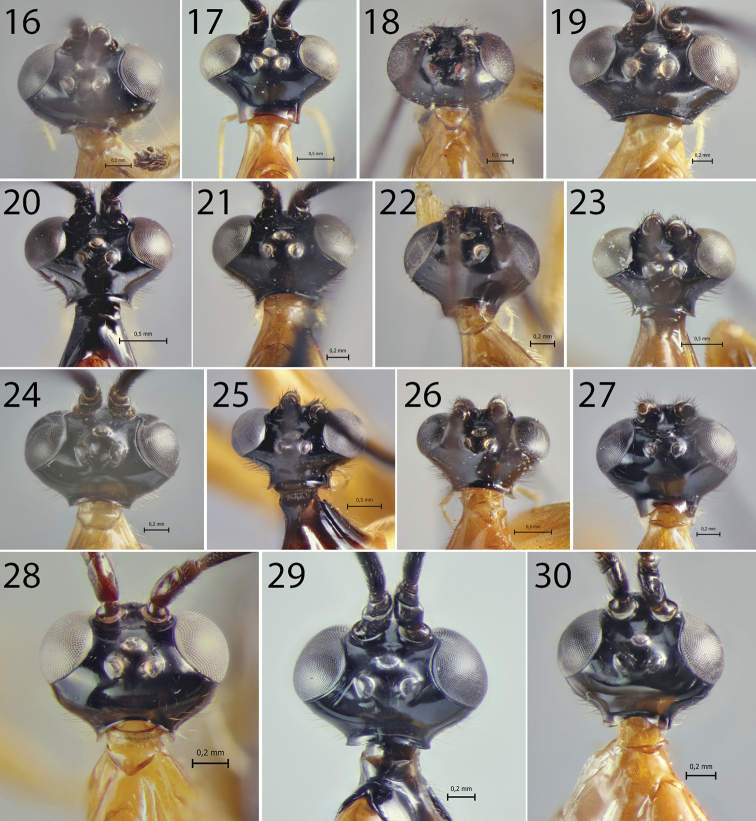
Heads of *Hymenoepimecis* spp. (females), dorsal view **16***H. andina* sp. nov. (holotype) **17***H. bicolor***18***H. castilloi* sp. nov. (holotype) **19***H. dolichocarinata* sp. nov. (holotype) **20***H. duckensis***21***H. ecuatoriana* sp. nov. (holotype) **22***H. kleini***23***H. longilobus* sp. nov. (holotype) **24***H. manauara***25***H. neotropica***26***H. pucallpina* sp. nov. (holotype) **27***H. rafaelmartinezi* sp. nov. (holotype) **28***H. ribeiroi***29***H. tedfordi***30***H. uberensis*.

**Figures 31–45.**
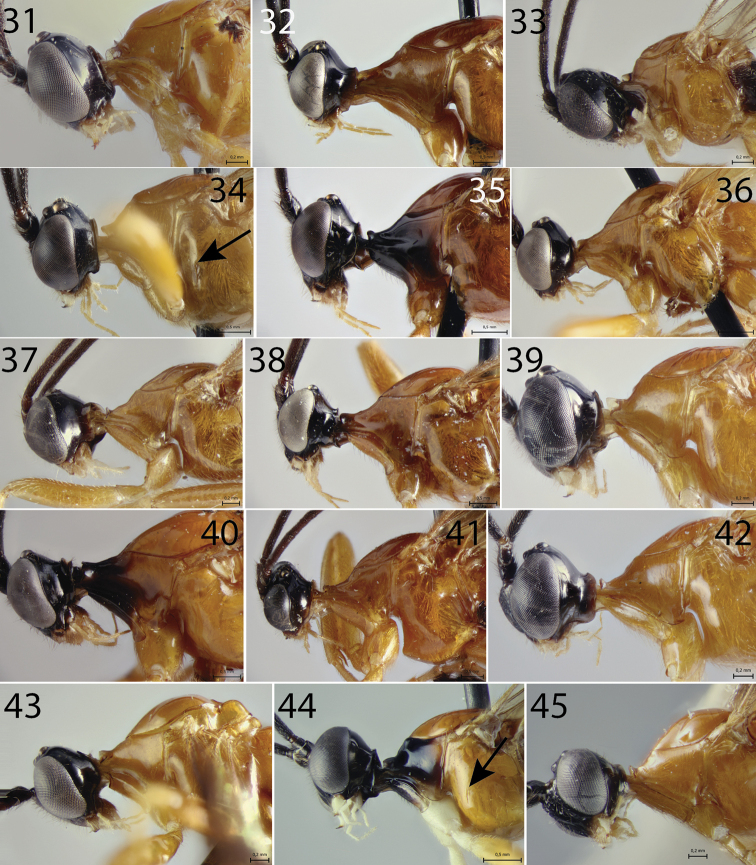
Head and part of mesosoma of *Hymenoepimecis* spp. (females), lateral view **31***H. andina* sp. nov. (holotype) **32***H. bicolor***33***H. castilloi* sp. nov. (holotype) **34***H. dolichocarinata* sp. nov. (holotype), arrow shows the epicnemial carina **35***H. duckensis***36***H. ecuatoriana* sp. nov. (holotype) **37***H. kleini***38***H. longilobus* sp. nov. (holotype) **39***H. manauara***40***H. neotropica***41***H. pucallpina* sp. nov. (holotype) **42***H. rafaelmartinezi* sp. nov. (holotype) **43***H. ribeiroi***44***H. tedfordi*, arrow shows the epicnemial carina **45***H. uberensis*.

**Figures 46–60.**
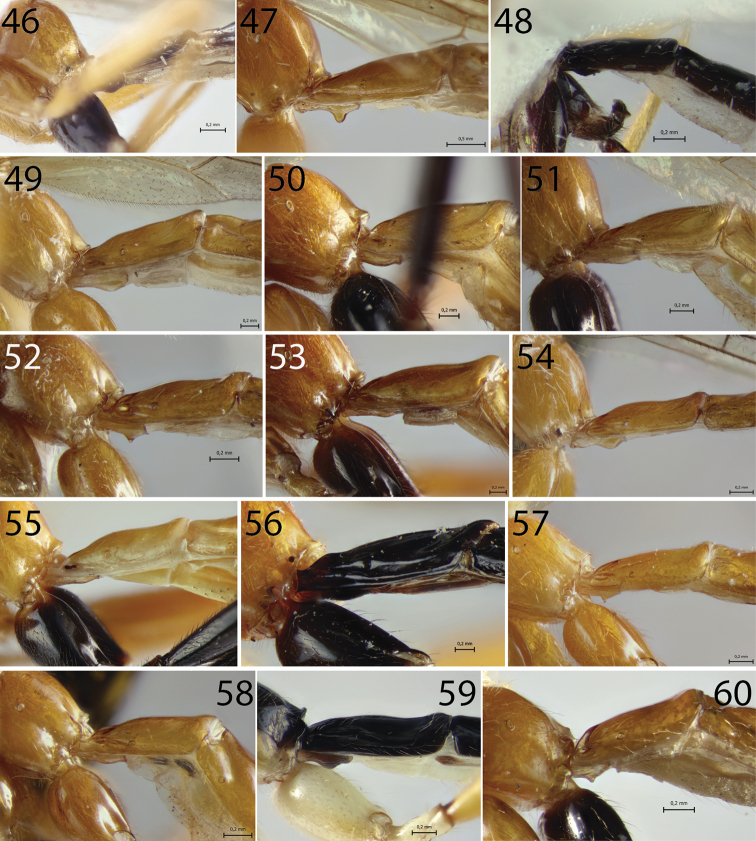
Sternite I of *Hymenoepimecis* spp. (females), lateral view **46***H. andina* sp. nov. (holotype) **47***H. bicolor***48***H. castilloi* sp. nov. (holotype) **49***H. dolichocarinata* sp. nov. (holotype) **50***H. duckensis***51***H. ecuatoriana* sp. nov. (holotype) **52***H. kleini***53***H. longilobus* sp. nov. (holotype) **54***H. manauara***55***H. neotropica***56***H. pucallpina* sp. nov. (holotype) **57***H. rafaelmartinezi* sp. nov. (holotype) **58***H. ribeiroi***59***H. tedfordi***60***H. uberensis*.

**Figures 61–66.**
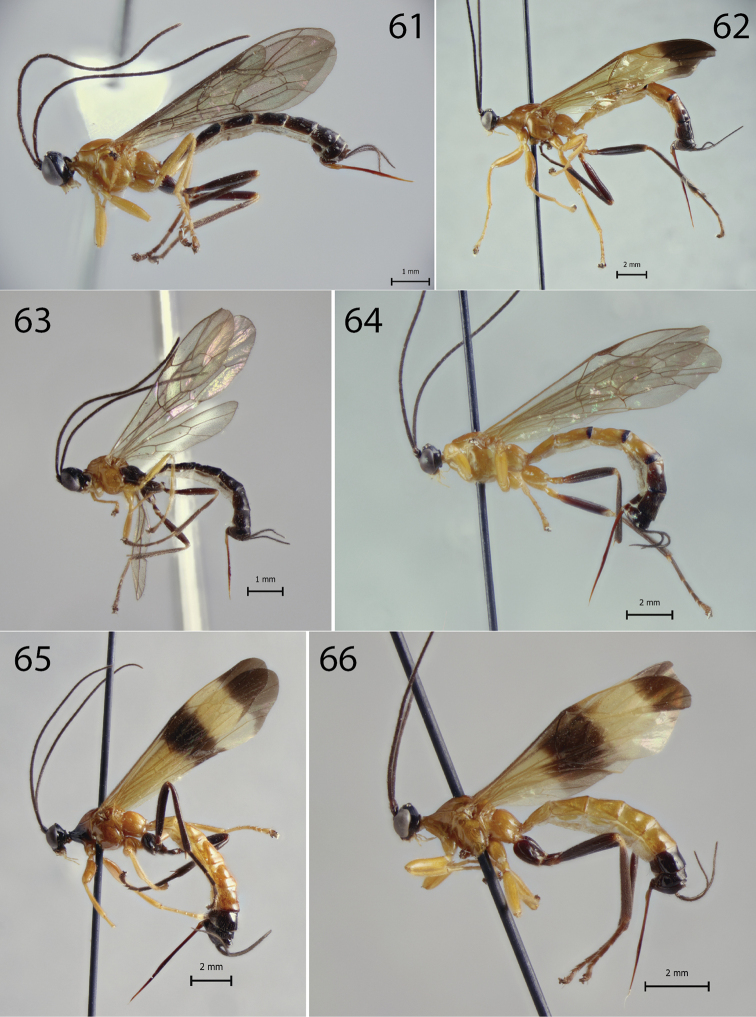
Habitus of *Hymenoepimecis* spp. (females), lateral view **61***H. andina* sp. nov. (holotype) **62**, *H. bicolor***63***H. castilloi* sp. nov. (holotype) **64***H. dolichocarinata* sp. nov. (holotype) **65***H. duckensis***66***H. ecuatoriana* sp. nov. (holotype).

Figures 1 , 16 , 31 , 46 , 61 , 76 , 97

#### Diagnosis.

This species can be distinguished from all other *Hymenoepimecis* by the combination of the following characters: 1) head, in dorsal view, with gena short and slightly convex behind eyes; 2) posterior ocelli separated from eyes by 0.7–0.8 times its own maximum diameter; 3) occipital carina only slightly projected and not curved upwards dorsally; 4) ovipositor 1.1–1.2 times as long as hind tibia; 5) metasoma darkish brown, with whitish lateral marks on anterior margins of tergites II–V.

#### Description.

**Female.** Body approx. [8.0] 7.0–8.5 mm; face [0.8] times as broad as high, smooth, slightly convex with few spaced bristles laterally; head in dorsal view, with gena short and slightly convex behind eyes; posterior ocelli separated from eyes by approx. [0.7] 0.7–0.8 times its own maximum diameter; occipital carina little projected and not curved upwards dorsally. Pronotum more or less long, smooth and polished, with distance from tegula to head greater than [0.5] 0.5–0.6 times distance from tegula to hind margin of propodeum, and in anterior part with opening pocket-like structure not reduced longitudinally; mesoscutum smooth and polished; scutellum, in profile, convex; mesopleuron smooth and polished, with anterodorsal and posterodorsal parts bearing sparse, fine setiferous punctures; metapleuron smooth and polished, with few sparse, fine setiferous punctures; propodeum smooth, polished, with sparse, fine setiferous punctures and with lateral longitudinal carina present only posteriorly. Fore wing approx. [6.0] 6.0–7.0 mm; cu-a more or less interstitial to the base of Rs&M; 2rs-m approx. [0.3] 0.3–0.5 times as long as abscissa of M between 2rs-m and 2m-cu; hind wing with abscissa of Cu1 meet cu-a equidistant between 1A and M. Hind leg with tibia + tarsus [0.6] times fore wing length; tarsal claw with more or less square basal lobe with apex of claw slightly overtaking the lobe. Metasoma slender; tergite I [1.7] 1.6–1.7 times as long as posteriorly width, centrally quite strongly convex with lateral carinae present only at extreme anterior end flanking the anterior concavity; sternite I with a low, rounded swelling posteriorly; tergite II approx. [1.4] 1.3–1.4 times as long as posteriorly width; tergites III and IV approx. [1.2] 1.2–1.3 times as long as posteriorly width; ovipositor [1.1] 1.1–1.2 times as long as hind tibia.

***Colour.*** Head black with apical margin of clypeus and mouthparts (except apex mandible black) yellowish; antenna blackish. Mesosoma orange. Fore and mid leg orange, the hind leg black. Wings hyaline slightly darkish; pterostigma brownish. Metasoma darkish brown, with whitish lateral marks on anterior margins of tergites II–V; ovipositor brownish with base and apex whitish, sheath blackish.

#### Male.

(Fig. 97). Similar to female in structure and colouration, but with body with 6.0–8.0 mm; face approx.1.1 times as width as high; posterior ocelli separated from eyes by 0.7–0.9 times its own maximum diameter. Fore wing 4.5–6.5 mm. Tarsal claw simple. Metasoma slender; tergite I 1.6 times as long as posteriorly width; tergite II approx.1.4 times as long as posteriorly width; tergites III and IV 1.2–1.3 times as long as posteriorly width.

#### Distribution.

Peru (Andes) (Fig. 107).

#### Biological notes.

Host unknown.

#### Etymology.

The specific name refers the locality of the specie type, in the Peruvian Andes, Cusco Peru.

#### Type material.

***Holotype*** ♀. Peru, Cusco, San Pedro, 13°02'58"S, 71°32'13"W, 1500 m., 20.ix.2007, Malaise trap (C. Castillo leg.), MUSM. ***Paratypes***: idem holotype, 4♂♂ and 2♀♀, MUSM; idem, but 23.vii.2007, 3♂♂, MUSM; idem, but 13°03'22"S, 71°32'55"W, 1520 m., 25.vii.2007, 1♀, MUSM; idem, but Cosñipata valley, 13°03'11"S, 71°32'08"W, 1302 m., 1♀, ZMUT; idem, but 13°03'23"S, 71°32'55"W, 24.x.2007, 1♀, ZMUT.

#### Comments.

*Hymenoepimecis andina* sp. nov. closely resembles *H. tedfordi* Gauld, 1991 and *Hymenoepimecis castilloi* sp. nov., mainly by having hyaline fore wing and darkish metasoma. It differs from both by having metasoma with whitish lateral marks on anterior margins of tergites II–V and propodeum orange (metasoma without whitish marks and propodeum black in other two species).

### Hymenoepimecis bicolor

Taxon classificationAnimaliaHymenopteraIchneumonidae

(Brullé, 1846)

8817CCF6-2623-5BB0-90C4-5A41D4A9C7AA

Figures 2 , 17 , 32 , 47 , 62 , 77 , 98

Epimecis bicolor Brullé, 1846: 113.
Hymenoepimecis bicolor (Brullé): Viereck, 1912: 149.

#### Diagnosis.

See Pádua et al. (2015).

#### Distribution.

Brazil, Ecuador** and Peru* (Fig. 108).

#### Biological notes.

Parasitoid of *Trichonephila clavipes* (Linnaeus, 1767) (as *Nephila clavipes*) (Araneae: Araneidae) (Gonzaga et al. 2010).

#### Material examined.

Ecuador: Dept. Orellana, Tiputini, 00°37'55"S, 76°08'39"W, a.s.l.: 220–250 m., 5.vii.1998, Fogging (T.L. Erwin et al. leg.), Lot #1894, 1♂, ZMUT; idem, but Onkonegare, 00°39'25"S, 76°27'10,8"W, a.s.l.: 216.3 m., 6.vii.1995, Lot #1129, 1♂, ZMUT. Peru: Dept. of Loreto, Iquitos area, Allpahuayo, 17.x–8.xi.2000, Malaise trap, clay (I.E. Sääksjärvi et al. leg.), APHI, H1/15, 1♀, ZMUT; idem, but 11–29.vi.2000, APHI, H1/8, 1♀, ZMUT; idem, but 14.ix–4.x.2000, APHI, H1/13, 1♀, ZMUT; idem, but 24.iii–16.iv.2000, white sand, APHI, G1/4, 1♀, ZMUT; idem, but 19.ix–4.x.2000, APHI, I1/13, 1♂, ZMUT; idem, but 24.i–20.ii.2000, APHI, G1/1, 1♀, ZMUT; idem, but 8–24.iii.2000, APHI, G2/3, 1♀, ZMUT; idem, but 22.v–11.vi.2000, APHI, 1♀ ZMUT; idem, but 20.ii–8.iii.2000 APHI, 1♀, ZMUT; idem, but iv.2011 (Gómez & Sääksjärvi leg.), 1♀, ZMUT; idem, but 30°57'49"S, 73°24'93"W, 1♀, ZMUT; idem, but 7–13.xi.2011, 1♀, ZMUT; idem, but 11–20.xi.2011, 1♀, ZMUT; idem, but 31.x–6.xi.2011, 1♀, ZMUT; idem, but 14–20.xi.2011, 1♀, ZMUT; 31.x–6.xi.2011, 1♀, ZMUT; idem, but 14–20.xi.2011, 1♀, ZMUT; idem, but 24–30.x.2011, 1♀, ZMUT; idem, but 3–9.x.2011, 1♀, ZMUT; 10–16.x.2011, 1♀, ZMUT; Alto Nanay, Albarenga north, 172 m., 18M 0532618E/ 9645753N, 17.xi.2008 (C. Castillo leg.), 1♂, ZMUT; idem, but Rio Copaluacu, 3°42'59"S, 75°26'0"W, 8.xii.2009, Malaise trap, 165 m. (L. Sulca leg.), 1♀, ZMUT; Dept. Madre de Díos, Los Amigos, 24–31.vii.2008, E: 380792, 164/N: 8610919,14, Malaise trap, a.s.l.: 280.5 (I. Gómez leg.), 1♂, ZMUT; idem, but Tambopata, Explore’s inn, 12°50'30"S, 69°17'31"W, 6.vii.2009, Malaise trap, 161 m. (M. Alvarado leg.), 1♀, ZMUT; Cusco, Reserva Comunal Amarakaeri, 12°55'S, 70°51'W, 333–884 m., 17.ix–14.xi.2010, Malaise trap (M. Vilchez & C. leg.), 1♀ and 2♂♂, ZMUT; idem, but La Convención, Echarate, San Martin Norte, 11°45'18.8"S, 72°42'26"W, 430 msnm, 10–14.xi.2010 (B. Medina & Z. Bravo leg.), 1♂, ZMUT; idem, but Pagoreni Camp., 465m., 11°47'05,4"S, 72°42'03"W, 25.ix.1998, Flight intercept trap, Camisea Project, 1♂, ZMUT; idem, but 23.ix.1998, 3♂♂, ZMUT; idem, but 5.v.1998, 2♂♂, ZMUT.

### Hymenoepimecis castilloi

Taxon classificationAnimaliaHymenopteraIchneumonidae

Pádua & Sääksjärvi sp. nov.

1E9DBE09-FD79-55CC-AC72-26BB89EB7A9D

http://zoobank.org/BE9BBA71-5809-4E5C-AA94-6C59F601E7B0

Figures 3 , 18 , 33 , 48 , 63 , 78

#### Diagnosis.

This species can be distinguished from all other *Hymenoepimecis* by the combination of the following characters: 1) head in dorsal view with gena short and slightly convex behind eyes; 2) posterior ocelli separated from eyes by approx. 0.7 times its own maximum diameter; 3) occipital carina only slightly projected and not curved upwards dorsally; 4) ovipositor 1.1 times as long as hind tibia; 5) mesosoma orange, with propleuron, metapleuron (except its posterior margin whitish), and propodeum darkish brown; 6) metasoma darkish brown.

#### Description.

**Female.** Body approx. [6.0] mm; face [0.9] times as broad as high (from supraclypeal suture to base of antenna), smooth, flat with few spaced bristles laterally; head in dorsal view, with gena short and slightly convex behind eyes; posterior ocelli separated from eyes by [0.7] times its own maximum diameter; occipital carina little projected and not curved upwards dorsally. Pronotum as long as high, smooth and polished, with distance from tegula to head greater than approx. [0.7] times distance from tegula to hind margin of propodeum, and in anterior part with opening pocket-like structure not reduced longitudinally; mesoscutum smooth and polished; scutellum, in profile, convex; mesopleuron smooth and polished, with posterodorsal part bearing sparse, fine setiferous punctures; metapleuron smooth and polished, with a few sparse, fine setiferous punctures; propodeum smooth, polished, with sparse, fine setiferous punctures and with lateral longitudinal carina present only posteriorly. Fore wing [5.0] mm; cu-a more or less interstitial to the base of Rs&M; 2rs-m approx. [0.3] times as long as abscissa of M between 2rs-m and 2m-cu; hind wing with abscissa of Cu1 meet cu-a equidistant between 1A and M. Hind leg with tibia + tarsus approx. [0.6] times fore wing length; tarsal claw with more or less square basal lobe with apex of claw slightly overtaking the lobe. Metasoma slender; tergite I [2.0] times as long as posteriorly width, centrally quite strongly convex with lateral carinae present only at extreme anterior end flanking the anterior concavity; sternite I with a low, rounded swelling posteriorly; tergite II approx. [1.4] times as long as posteriorly width; tergites III–IV approx. [1.1] times as long as posteriorly width; ovipositor [1.1] times as long as hind tibia.

***Colour.*** Head black with apical margin of clypeus and mouthparts (except apex of mandible) yellowish; antenna brownish. Mesosoma orange, with propleuron, metapleuron (except its posterior margin whitish), and propodeum darkish brown. Fore and mid leg orange, the hind leg darkish brown. Wings hyaline; pterostigma brownish. Metasoma darkish brown; ovipositor brownish with base and apex whitish, sheath brownish.

#### Male.

Unknown.

#### Distribution.

Peru (Andes) (Fig. 108).

#### Biological notes.

Host unknown.

#### Etymology.

This species is named in honour of Carol Castillo, collector of the type specimen. Carol is a Peruvian entomologist and has studied Darwin wasps in the tropical Andes. She has discovered and/or described a large number of new species.

#### Type material.

***Holotype*** ♀. Peru, Cusco, Cosñipata valley, San Pedro, 13°03'23"S, 71°32'55"W, 1520 m., 24.x.2007, Malaise trap (C. Castillo leg.), MUSM.

#### Comments.

*Hymenoepimecis castilloi* sp. nov. closely resembles *H. tedfordi* Gauld, 1991 and *Hymenoepimecis andina* sp. nov., mainly by having fore wing hyaline and metasoma darkish. It differs from *H. tedfordi* by having mesosoma orange, with propleuron, metapleuron (except its posterior margin whitish), and propodeum darkish brown, hind legs entirely brownish and occipital carina short (mesosoma orange, with propleuron, pronotum, dorsal half of metapleuron, and propodeum blackish hind leg with coxa whitish and femur, tibia, and tarsus orange and occipital carina long, in *H. tedfordi*). It differs from *H. andina* sp. nov. by having mesosoma orange, with propleuron, metapleuron (except its posterior margin whitish) and propodeum darkish brown and metasoma darkish brown without whitish bands anteriorly (propodeum orange and metasoma darkish brown, with whitish lateral marks on anterior margins of tergites II–V in *H. andina* sp. nov.).

### Hymenoepimecis dolichocarinata

Taxon classificationAnimaliaHymenopteraIchneumonidae

Pádua & Sääksjärvi sp. nov.

24AC9172-9382-59ED-9F4C-F733BB59E5C3

http://zoobank.org/66F48B80-3F42-4EDB-8B3B-DA1D9DB4ABD0

Figures 4 , 19 , 34 , 49 , 64 , 79 , 99

#### Diagnosis.

This species can be distinguished from all other *Hymenoepimecis* by the combination of the following characters: 1) epicnemial carina present ventrally, extending to the level of the lower corner of the pronotum laterally; 2) wings slightly yellowish hyaline; 3) margin of the gena flat behind the eyes; 4) metasoma orange, with posterior margins of tergites II–IV narrowly black, tergites V+ black.

#### Description.

**Female.** Body approx. [13.0] 11.5–13.0 mm; face [1.0] 0.9–1.0 times as broad as high, smooth, slightly convex with few spaced bristles; head in dorsal view, with margin of gena flat behind eyes; posterior ocelli separated from eyes by approx. [0.6] 0.5–0.6 times its own maximum diameter; occipital carina little projected, slightly curved upwards in the mediodorsal part. Pronotum more or less long, smooth and polished, with distance from tegula to head greater than [0.6] 0.5–0.6 times distance from tegula to hind margin of propodeum, and in anterior part with opening pocket-like structure not reduced longitudinally; mesoscutum smooth and polished; scutellum, in profile, convex; mesopleuron smooth and polished, with anterodorsal and posterodorsal parts bearing sparse, fine setiferous punctures; epicnemial carina present ventrally, extending until reaching the level of lower corner of pronotum laterally; metapleuron smooth and polished, rather uniformly covered with sparse, fine setiferous punctures. Fore wing approx. [10.0] 9.0–11.0 mm; cu-a interstitial to the base Rs&M; 2rs-m approx. [0.6] times as long as abscissa of M between 2rs-m and 2m-cu; hind wing with abscissa of Cu1 meeting cu-a equidistant between M and 1A. Hind leg with tibia + tarsus [0.6] 0.55–0.6 times the fore wing length; tarsal claw with more or less square lobe, with apex slightly overtaking the lobe. Metasoma slender; tergite I [1.6] 1.5–1.6 times as long as posteriorly width, centrally quite strongly convex with lateral carinae present only at extreme anterior end flanking the anterior concavity; sternite I with a low, rounded swelling posteriorly; tergite II [1.4] 1.3–1.4 times as long as posteriorly width; tergites III and IV approx. [1.5] 1.2–1.5 times as long as posteriorly width; ovipositor [1.45] 1.3–1.5 times as long as hind tibia.

***Colour.*** Head black; clypeus yellowish, with base slightly black; mouthparts yellowish, with apex mandible black; antenna brownish. Mesosoma orange. Fore and mid leg orange, the hind leg orange, with femur, tibia, and tarsus brownish. Wings slightly yellowish hyaline; pterostigma brown. Metasoma orange, with tergites II–V with lateral marks on posterior margins black and tergites V+ brownish; ovipositor and sheath brown.

#### Male.

(Fig. 99). Similar to female in structure and colouration, but with body with 5.0–7.0 mm; face 0.9–1.1 times as broad as high; head in dorsal view, with margin of gena short, slightly convex behind eyes; posterior ocelli separated from eyes by approx. 0.8 times its own maximum diameter; occipital carina not projected. Fore wing 4.0–6.0 mm; cu-a more or less interstitial to the base Rs&M; 2rs-m 0.3–0.4 times as long as abscissa of M between 2rs-m and 2m-cu. Tarsal claw simple. Metasoma slender; tergite I 1.6–1.7 times as long as posteriorly width; tergite II approx. 1.3 times as long as posteriorly width; tergites III and IV 1.2 times as long as posteriorly width.

#### Variation.

Tergite V black with anterior margin orange or apical half orange and basal half-black. Three females from French Guiana (Saül city) presented margin of gena slightly convex. We think they are conspecific, but we are not treating them as paratypes.

**Figures 67–71.**
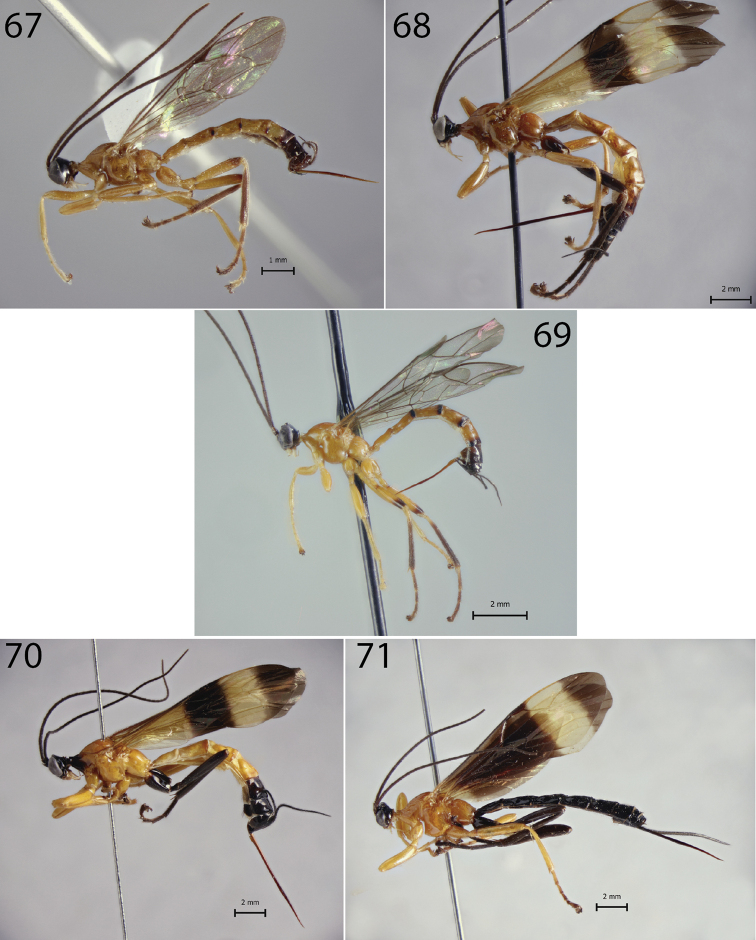
Habitus of *Hymenoepimecis* spp. (females), lateral view **67***H. kleini***68***H. longilobus* sp. nov. (holotype) **69***H. manauara***70***H. neotropica***71***H. pucallpina* sp. nov. (holotype).

#### Distribution.

Ecuador**, French Guiana and Peru (Fig. 107).

#### Biological notes.

Host unknown.

#### Etymology.

The specific name refers to the long epicnemial carina, reaching the level of the lower corner of the pronotum laterally.

#### Type material.

***Holotype*** ♀. Peru, Dept. of Loreto, Iquitos area, Allpahuayo, APHI, 29.i–20.ii.2000, Malaise trap I1, (I.E. Sääksjärvi et al. leg.), MUSM. ***Paratypes***: Peru: idem holotype, 1♀, ZMUT; Dept. of Loreto, Iquitos area, Allpahuayo, 20.ii–8.iii.2000, white sand, Malaise trap (Sääksjärvi et al. leg.), APHI, G2/2, 1♀, MUSM; idem, but 30°57'84"S, 73°25'39"W, 5–11.xii.2011 (Gómez & Sääksjärvi leg.), 1♀, ZMUT; idem, but H2 (16), 21.xii.2000, 1♀, ZMUT; idem, but J1, 1.xii.2000, 1♀, ZMUT. Ecuador: Dept. Orellana, Onkonegare, 00°39'25,7"S, 76°27'10,8"W, a.s.l.: 216 m., 08.ii.1996, Fogging (T.L. Erwin leg.), Lot #1473, 1♂, ZMUT; idem, but 30.ix.1996, Lot #1677, 1♂, ZMUT.

#### Other material.

French Guiana, Saül, 27.xii or viii.2011 [sic], Malaise trap (without collector), 1♀, ZMUT; idem, but 7.v.2012, 1♀, ZMUT; idem, but 13.xii.2011, 1♀, ZMUT.

#### Comments.

*Hymenoepimecis dolichocarinata* sp. nov. closely resembles *H. japi* Sobczak, Loffredo, Penteado-Dias & Gonzaga, 2009, *H. sooretama* Sobczak, Loffredo, Penteado-Dias & Gonzaga, 2009 and *H. manauara* Pádua & Oliveira mainly by having weak black lateral marks on posterior margins of tergites II–V and by having sternite I with a low, rounded swelling posteriorly, but it differs from them mainly by having epicnemial carina present (absent in all other mentioned species).

### Hymenoepimecis duckensis

Taxon classificationAnimaliaHymenopteraIchneumonidae

Pádua & Onody, 2015

62A5CFC2-A0A8-57EC-AD1B-8090D03CE75A

Figures 5 , 20 , 35 , 50 , 65 , 80 , 100

Hymenoepimecis duckensis Pádua & Onody, 2015: 181.

#### Diagnosis.

See Pádua et al. (2015).

#### Distribution.

Brazil, Ecuador**, French Guiana* and Peru* (Fig. 109).

#### Biological notes.

Host unknown.

#### Material examined.

French Guiana: M. de Kaw, Patawa (PM), ii.2003 (O. Morvan leg.), 1♂ and 1♀, ZMUT; idem, but 3.iii.2003, 1♂ and 1♀, ZMUT. Ecuador: Dept. Orellana, Onkonegare, 00°39'25,7"S, 76°27'10,8"W, a.s.l.: 216 m., 4.x.1996, Fogging, Lot #1758 (T.L. Erwin leg.), 1♂, ZMUT; idem, but 6.x.1995, Lot #1217, 1♂, ZMUT; idem, but Tiputini, 00°37'55"S, 76°08'39"W, a.s.l.: 220–250 m., 28.x.1998, Lot #1955, 1♀, ZMUT; idem, but 00°39'25,7"S, 76°27'10,8"W, 216.3 m., 5.ii.1996, Lot #1422, 1♂, ZMUT; Napo province, Yasuni National Park, PUCE, 00°38'S, 76°36'W, 20.xi.1998, Malaise trap (T. Pape & B. Viklund leg.), 1♂, ZMUT. Peru: Loreto, Alto Nanay, Albarenga north, 195 m., 18M 0530961E/9646100N, 20.xi.2008 (C. Castillo leg.), 1♂, ZMUT; idem, but Qda. Pucacuro, Bosque de terraza media, 18M 0501611E/9726184N, 173 m., 24.x.2008, Malaise trap (M. Vilchez leg.), 1♀, ZMUT; idem, but Iquitos area Allpahuayo, 29.vi–16.vii.2000, Malaise trap, white sand (I.E. Sääksjärvi & et al.), APHI, E1/9, 1♀, ZMUT; idem, but 30°57'49"S, 73°24'93"W, 3–9.x.2011 (Gómez & Sääksjärvi leg.), 1♀, ZMUT; idem, but clay (I.E. Sääksjärvi et al. leg.), 22.v–11.vi.2000, J1 (7), 1♀, ZMUT.

**Figures 72–75.**
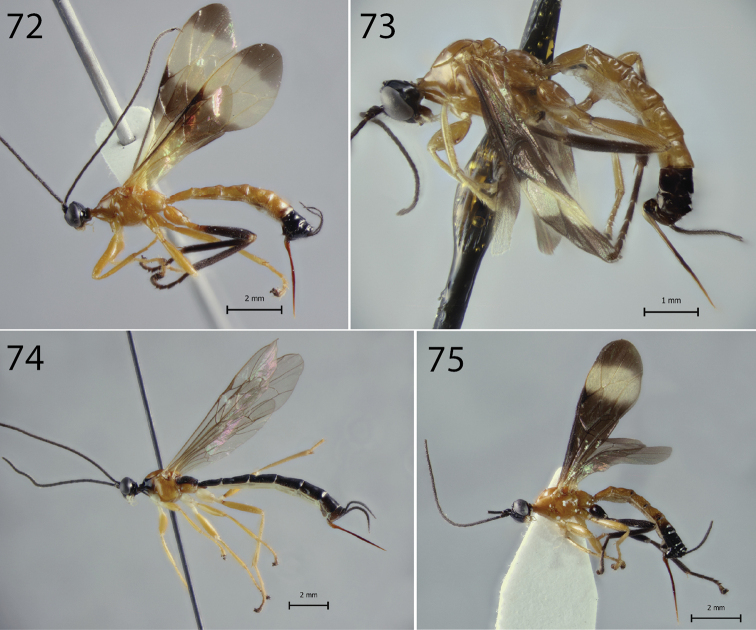
Habitus of *Hymenoepimecis* spp. (females), lateral view **72***H. rafaelmartinezi* sp. nov. (holotype) **73***H. ribeiroi***74***H. tedfordi***75***H. uberensis*.

### Hymenoepimecis ecuatoriana

Taxon classificationAnimaliaHymenopteraIchneumonidae

Pádua & Sääksjärvi sp. nov.

837C0D99-1EB8-50E5-8C10-6F1F5B60BCDF

http://zoobank.org/D27F8C9C-3B30-4690-A01C-79723F7B1A11

Figures 6 , 21 , 36 , 51 , 66 , 81

#### Diagnosis.

This species can be distinguished from all other *Hymenoepimecis* by the combination of the following characters: 1) fore wing hyaline yellowish, with two blackish bands; 2) pronotum orange; 3) female with tarsal claw with more or less square lobe, and with apex overtaking the lobe; 4) female with ovipositor 1.2 times as long as hind tibia.

**Figures 76–90.**
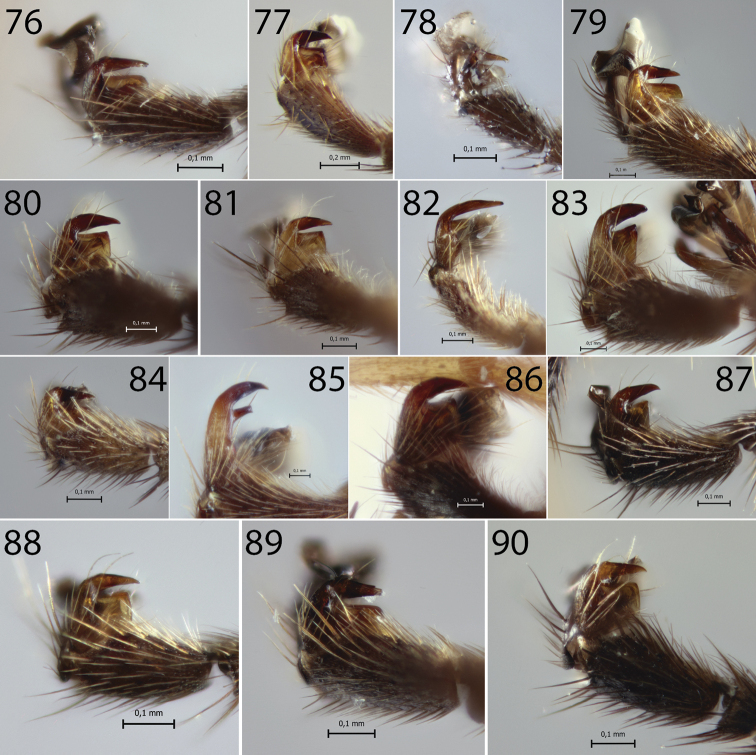
Tarsal claws of *Hymenoepimecis* spp. (females), lateral view **76***H. andina* sp. nov. (holotype) **77***H. bicolor***78***H. castilloi* sp. nov. (holotype) **79***H. dolichocarinata* sp. nov. (holotype) **80***H. duckensis***81***H. ecuatoriana* sp. nov. (holotype) **82***H. kleini***83***H. longilobus* sp. nov. (holotype) **84***H. manauara***85***H. neotropica***86***H. pucallpina* sp. nov. (holotype) **87***H. rafaelmartinezi* sp. nov. (holotype) **88***H. ribeiroi***89***H. tedfordi***90***H. uberensis*.

#### Description.

**Female.** Body [9.0] mm; face [1.0] times as broad as high, smooth, slightly convex with few spaced bristles; head in dorsal view, with gena strongly narrowed behind eyes; posterior ocelli separated from eyes by approx. [1.0] times its own maximum diameter; occipital carina projected and weakly reduced and curved upwards dorsally. Pronotum long, smooth and polished, with distance from tegula to head approx. [0.8] times distance from tegula to hind margin of propodeum, and in anterior part with opening pocket-like structure not reduced longitudinally; mesoscutum smooth and polished; scutellum, in profile, convex; mesopleuron smooth and polished, with anterodorsal and posterodorsal parts bearing sparse, fine setiferous punctures; metapleuron smooth and polished, rather uniformly covered with sparse, fine setiferous punctures; propodeum smooth, polished, with sparse, fine setiferous punctures and with lateral longitudinal carina present only posteriorly. Fore wing [7.0] mm; cu-a more or less interstitial to the base of Rs&M; 2rs-m [0.5] times as long as abscissa of M between 2rs-m and 2m-cu; hind wing with abscissa of Cu1 meeting cu-a equidistant between M and 1A. Hind leg with tibia + tarsus [0.6] times the fore wing length; tarsal claw with more or less square lobe, with apex of claw overtaking the lobe. Metasoma slender; tergite I [1.4] times as long as posteriorly width, centrally quite strongly convex with lateral carinae present only at extreme anterior end flanking the anterior concavity; sternite I with a low, rounded swelling posteriorly; tergite II approx. [1.2] times as long as posteriorly width; tergites III and IV approx. [1.1] times as long as posteriorly width; ovipositor [1.2] times as long as hind tibia.

***Colour.*** Head black; clypeus black with apex yellowish; mouthparts yellowish, with apex mandible black; antenna brownish. Mesosoma orange. Fore and mid leg orange, the hind entirely blackish brown. Fore wing hyaline yellowish, with apex blackish and with a blackish preapical band; pterostigma with basal half black and apical half yellow; hind wing with slightly blackish band in median part. Metasoma orange, with tergites VI+ black; ovipositor and sheath brownish.

#### Male.

Unknown.

#### Distribution.

Ecuador** (Fig. 109).

#### Biological notes.

Host unknown.

#### Etymology.

The specific name refers to Ecuador.

#### Type material.

***Holotype*** ♀. Ecuador, Dept. Orellana, Yasuni, 00°37'55"S, 76°08'39"W, a.s.l.: 220–250 m., 5.ii.1999, Fogging, Lot #2086 (T.L. Erwin leg.), ZMUT.

**Figures 91–96.**
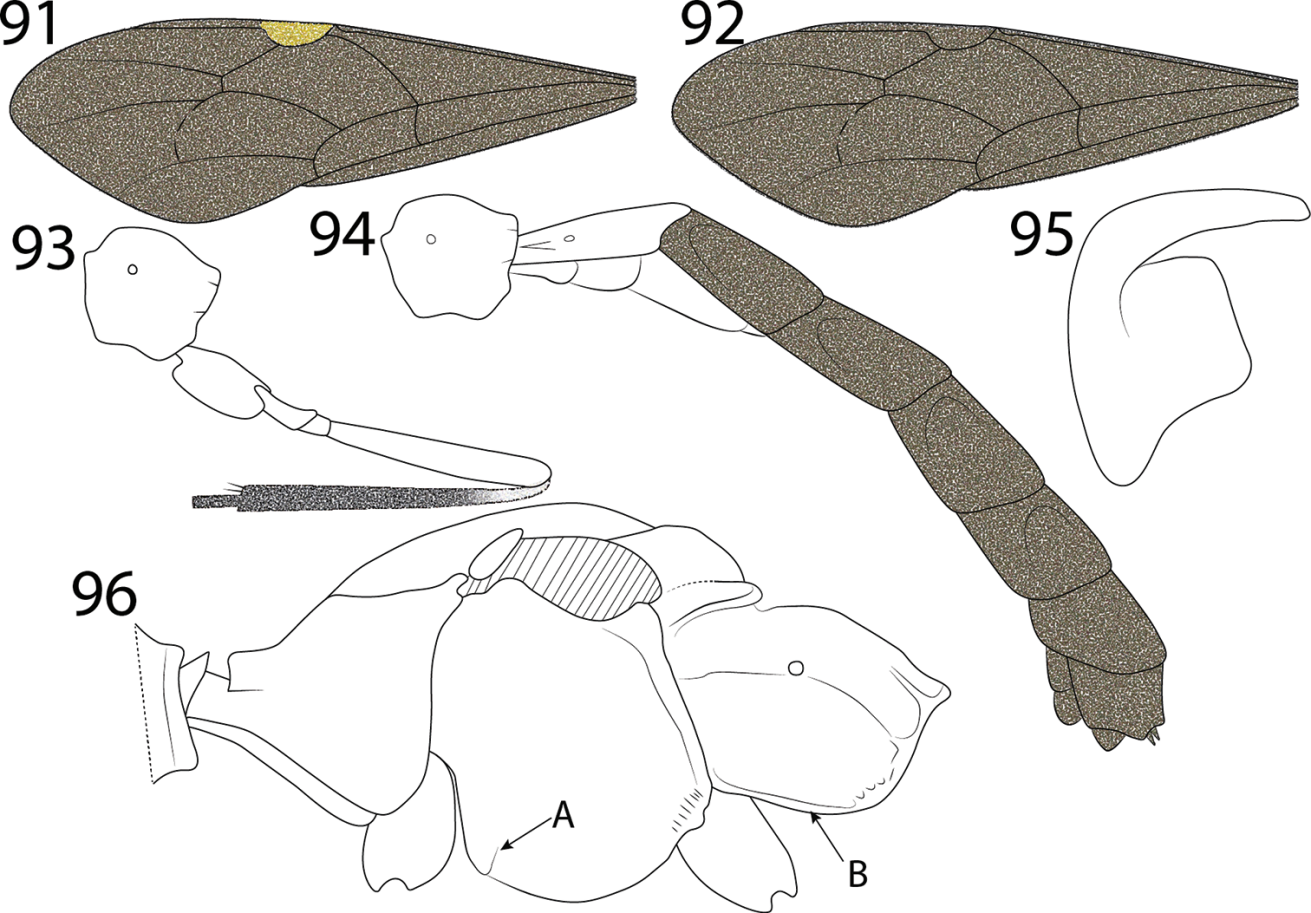
*Hymenoepimecis* spp. **91, 92** fore wing, showing colour pattern. (**91**) *H. veranii* (**92**) *H. argyraphaga***93***H. robertsae*, propodeum and hind leg, showing colour pattern, lateral view **94***H. heteropus*, propodeum and metasoma, showing colour pattern, lateral view **95***H. amazonensis* (female), tarsal claw, lateral view **96***H. argyraphaga*, mesosoma, lateral view: Arrow “A” shows epicnemial carina and arrow “B” shows submetapleural carina.

#### Comments.

*Hymenoepimecis ecuatoriana* sp. nov. closely resembles *H. neotropica* (Brues & Richardson, 1913), *Hymenoepimecis longilobus* sp. nov. and *H. duckensis* Pádua & Onody, 2015 mainly by having the fore wing yellowish hyaline with two blackish bands, metasoma orange with last tergites black and face without a sculptured, longitudinal carina in the middle part of face. It differs from the first and second congeneric species by having tarsal claw with a more or less square lobe (tarsal claw with a preapical tooth, in *H. neotropica* and lobe longitudinally elongated in *H. longilobus* sp. nov.), and from the last species by having the ovipositor < 1.3 times as long as hind tibia (> 1.5 times as long as hind tibia in *H. duckensis*).

### Hymenoepimecis kleini

Taxon classificationAnimaliaHymenopteraIchneumonidae

Pádua & Sobczak, 2015

91E98115-986E-58AF-9D42-EBB89B595B53

Figures 7 , 22 , 37 , 52 , 67 , 82 , 101

Hymenoepimecis kleini Pádua & Sobczak, 2015: 183.

#### Diagnosis.

See Pádua et al. (2015).

#### Male.

(Fig. 101). Similar to female in structure and colouration, but with hind leg black, except coxa, with basal part orange; body approx. 8.0 mm; face a approx. 1.1 times as broad as high; posterior ocelli separated from eyes by approx. 0.8 times its own maximum diameter. Fore wing approx. 6.0 mm; 2rs-m approx. 0.2 times as long as abscissa of M between 2rs-m and 2m-cu. Tarsal claw simple. Metasoma slender; tergite I approx. 1.6 times as long as posteriorly width; tergite II approx. 1.4 times as long as posteriorly width; tergites III and IV approx. 1.3 times as long as posteriorly width.

**Figures 97–102.**
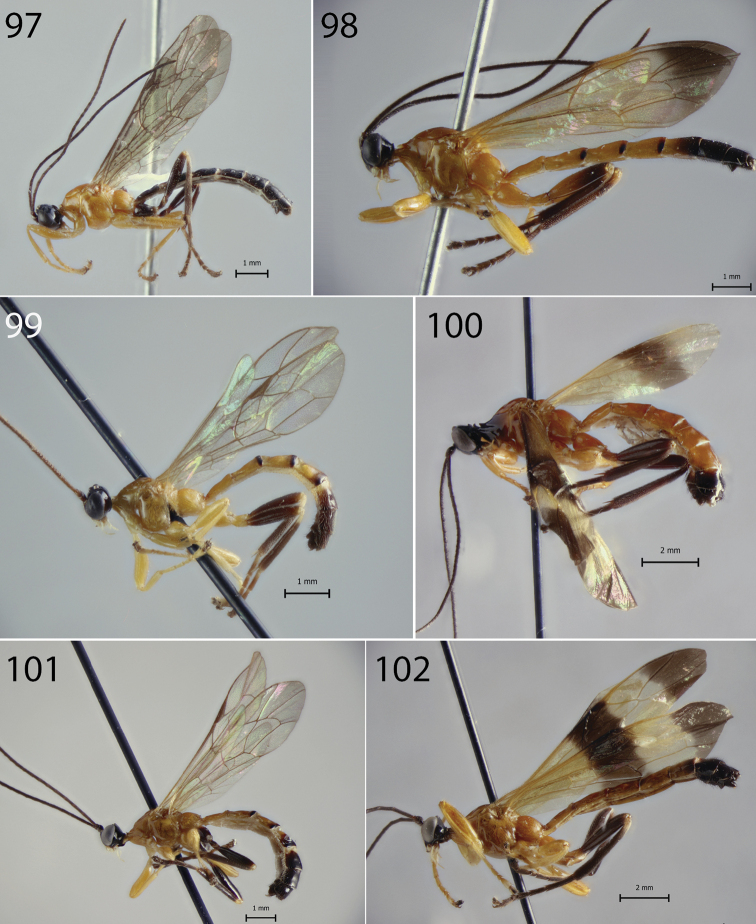
Habitus of *Hymenoepimecis* spp. (males), lateral view **97***H. andina* sp. nov. (paratype) **98***H. bicolor***99***H. dolichocarinata* sp. nov. (paratype) **100***H. duckensis***101***H. kleini***102***H. longilobus* sp. nov. (paratype).

#### Distribution.

Brazil, Ecuador** and Peru* (Fig. 107).

#### Biological notes.

Host unknown.

#### Material examined.

Ecuador, Dept. Orellana, Onkonegare, 00°39'25,7"S, 76°27'10,8"W, a.s.l.: 216.3 m., 21.vi.1996, Fogging, Lot # 1543 (T.L. Erwin et al. leg.), 1♂, ZMUT. Peru: Cusco, Cashiariari, 11°52'S, 72°39'W, 24.xi.1997, 690 m. (S. Cordova leg.), 1♀, ZMUT.

### Hymenoepimecis longilobus

Taxon classificationAnimaliaHymenopteraIchneumonidae

Pádua & Sääksjärvi sp. nov.

ED0FB537-ED74-5651-A5A4-B38F6F77279E

http://zoobank.org/8FFC81FF-D55A-4C66-A274-8EA5EAA3164F

Figures 8 , 23 , 38 , 53 , 68 , 83 , 102

#### Diagnosis.

This species can be distinguished from all other *Hymenoepimecis* by the combination of the following characters: 1) fore wing hyaline yellowish, with two blackish bands; 2) pronotum orange (Peruvian specimens with anterior margin of the pronotum blackish); 3) female with tarsal claw with a longitudinally elongated lobe, and with apex overtaking the lobe; 4) female with ovipositor 1.5–1.6 times as long as hind tibia.

#### Description.

**Female.** Body approx. [15.0] 12.0–15.0 mm; face [1.1] 1.0–1.1 times as broad as high, smooth, slightly convex with few spaced bristles; head in dorsal view, with gena strongly narrowed behind eyes; posterior ocelli separated from eyes by approx. [0.9] 0.8–1.0 times its own maximum diameter; occipital carina projected and curved upwards dorsally. Pronotum long, smooth and polished, with distance from tegula to head greater than approx. [0.7] 0.6–0.7 times distance from tegula to hind margin of propodeum, and in anterior part with opening pocket-like structure not reduced longitudinally; mesoscutum smooth and polished; scutellum, in profile, convex; mesopleuron smooth and polished, with anterodorsal and posterodorsal parts bearing sparse, fine setiferous punctures; metapleuron smooth and polished, rather uniformly covered with sparse, fine setiferous punctures; propodeum smooth, polished, with sparse, fine setiferous punctures and with lateral longitudinal carina present only posteriorly. Fore wing approx. [13.0] 9.0–13.0 mm; cu-a interstitial to the base of Rs&M; 2rs-m [0.55] 0.5–0.6 times as long as abscissa of M between 2rs-m and 2m-cu; hind wing with abscissa of Cu1 meeting cu-a equidistant between M and 1A. Hind leg with tibia + tarsus [0.6] times the fore wing length; tarsal claw with longitudinally elongated lobe, with apex of claw overtaking the lobe. Metasoma slender; tergite I [1.6] times as long as posteriorly width, centrally quite strongly convex with lateral carinae present only at extreme anterior end flanking the anterior concavity; sternite I with a low, rounded swelling posteriorly; tergite II approx. [1.2] 1.2–1.4 times as long as posteriorly width; tergites III and IV approx. [1.1] 1.1–1.3 times as long as posteriorly width; ovipositor approx. [1.6] 1.5–1.6 times as long as hind tibia.

**Figures 103–106.**
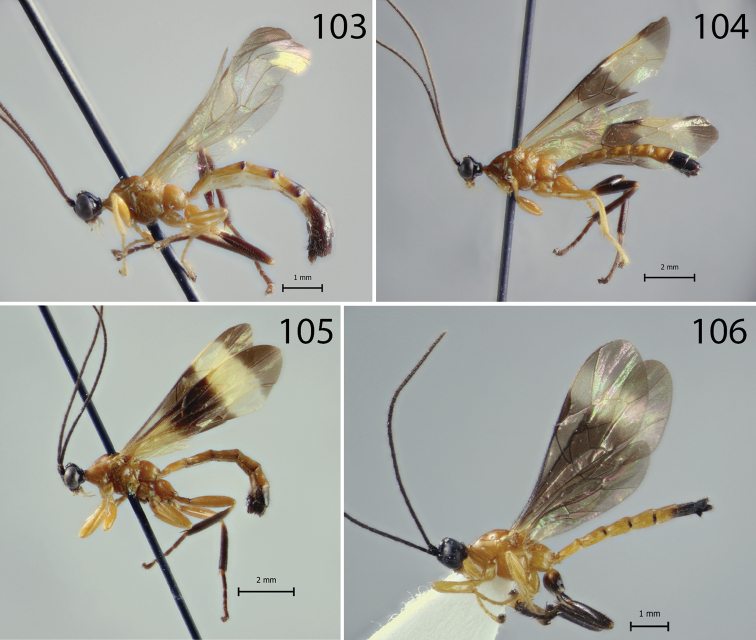
Habitus of *Hymenoepimecis* spp. (males), lateral view **103***H. manauara***104***H. rafaelmartinezi* sp. nov. (paratype) **105***H. ribeiroi***106***H. uberensis*.

***Colour.*** Head black; clypeus black with apex yellowish; mouthparts yellowish, with apex mandible black; antenna brownish. Mesosoma orange. Fore and mid leg orange, the hind entirely blackish brown except base of coxa orange. Fore wing hyaline yellowish, with apex blackish and with a blackish preapical band; pterostigma with basal half black and apical half yellow; hind wing with slightly blackish band in median part. Metasoma orange, with posterior margin black in tergite V and tergites VI+ black; ovipositor and sheath brownish.

#### Male.

(Fig. 102). Similar to female in structure and colouration, but with hind coxa orange body with 10.5–12.0 mm; face 1.0 times as broad as high; posterior ocelli separated from eyes by 0.8–1.0 times its own maximum diameter. Fore wing 8.0–10.0 mm; 2rs-m 0.3–0.5 times as long as abscissa of M between 2rs-m and 2m-cu. Tarsal claw simple. Metasoma slender; tergite I 1.6–1.7 times as long as posteriorly width; tergite II 1.2–1.5 times as long as posteriorly width; tergites III and IV 1.0–1.3 times as long as posteriorly width.

#### Variation.

The Peruvian specimens have anterior margin of pronotum blackish. Some specimens have tergite V entirely black or black with anterior margin orange; hind coxa orange with basal part blackish.

#### Distribution.

Ecuador**, French Guiana* and Peru* (Fig. 110).

#### Biological notes.

Host unknown.

#### Etymology.

The specific name refers the longitudinally elongated lobe in tarsal claw of the females.

#### Type material.

***Holotype*** ♀. French Guiana, M. de Kaw, Patawa, ix.2003, (J. Cerda leg.), ZMUT. ***Paratypes***: French Guiana: idem holotype, but vii.2003, 1♀, ZMUT; idem, but xi.2001, 1♂ and 1♀, ZMUT; idem, but xii.2001, 1♀, ZMUT; idem, but ii.2003 (O. Morvan leg.), 1♂, ZMUT. Ecuador: Dept. Orellana, Onkonegare, 00°39'25,7"S, 76°27'10,8"W, a.s.l.: 216 m., 23.vi.1996, Fogging, Lot #1616 (T.L. Erwin leg.), 1♂, ZMUT. Peru: Dept. of Loreto, Iquitos area, Allpahuayo, 22.ii–7.iii.2000, white sand, Malaise trap (Sääksjärvi et al. leg.), APHI, E1/2, 1♀, ZMUT; idem, but 14.ix–4.x.2000, clay, APHI, H1/13, 1♀, ZMUT; idem, but G1(15), 8.xi.2000, 1♀, ZMUT.

#### Comments.

*Hymenoepimecis longilobus* sp. nov. closely resembles *H. neotropica* (Brues & Richardson, 1913), *Hymenoepimecis ecuatoriana* sp. nov. and *H. duckensis* Pádua & Onody, 2015 mainly by having the fore wing yellowish hyaline with two blackish bands and metasoma orange with last tergites black. It differs from all of these species by having tarsal claw with a longitudinally elongated lobe (tarsal claw with a preapical tooth in *H. neotropica* and a more or less square lobe in *H. ecuatoriana* sp. nov. and *H. duckensis*).

### Hymenoepimecis manauara

Taxon classificationAnimaliaHymenopteraIchneumonidae

Pádua & Oliveira, 2015

7F63EEAB-6C76-5402-B66D-DA86E3385597

Figures 9 , 24 , 39 , 54 , 69 , 84 , 103

Hymenoepimecis manauara Pádua & Oliveira, 2015: 185.

#### Diagnosis.

See Pádua et al. (2015).

#### Distribution.

Brazil, French Guiana*, Ecuador** and Peru* (Fig. 111).

#### Biological notes.

Parasitoid of *Leucauge henryi* Mello-Leitão, 1940 (Araneae: Tetragnathidae) (Pádua et al. 2016).

#### Material examined.

French Guiana, M. de Kaw, Patawa, xi.2002 (PM) (J. Cerda leg.), 1♀, ZMUT; idem, but iii.2003, Malaise trap, 1♀, ZMUT; idem, but i.2002, 1♀, ZMUT; idem, but x.2001, 1♀, ZMUT; idem, but xi.2001, 2♀♀, ZMUT; idem, but iii.2002, 2♀♀ [one without head], ZMUT; idem, but ix.2003, 2♀♀, ZMUT; idem, but pk 35, x.2002, 1♀, ZMUT; idem, but ii.2003 (O. Morvan leg.), 1♀, ZMUT; Kourou, piste Soumourou, 2–19.iv.2002 (D. Faure leg.), 1♀, ZMUT; idem, but 12.v–10.vi.2002, 1♀, ZMUT; Montagne des Chevaux, 4.ix.2011 (SLAM leg.), 1♂ and 1♀, ZMUT. Ecuador: Napo province, Yasuni National Park, 00°38'S, 76°36'W, PUCE, Malaise trap, 20.xi.1998, Malaise trap (T. Pape & B. Viklund leg), NHRS, 4♀♀, ZMUT; Dept. Orellana, Tiputini, 00°37'55"S, 76°08'39"W, a.s.l.: 220–250 m., 9.ii.1999, Fogging, Lot #2001 (T.L. Erwin et al. leg.), 1♂, ZMUT; idem, but Yasuni, 00°37'55"S, 76°08'39"W, 5.vii.1998, Lot #1896, 1♂, ZMUT; idem, but 21.x.1998, Lot #1987, 1♂, ZMUT; idem, but Onkonegare, 00°39'25,7"S, 76°27'10,8"W, a.s.l.: 216 m., 6.x.1995, 1♀, ZMUT; idem, but 4.ii.1996, Lot #1416, 1♀, ZMUT; idem, but 9.ii.1995, Lot #984, 1♀, ZMUT; idem, but 30.ix.1996, Lot #1678, 1♀, ZMUT; idem, but 6.vii.1995, Lot #1130, 1♂, ZMUT; idem, but 8.x.1995, Lot #1263, 1♀, ZMUT. Peru: Dept. Madre de Dios, Los Amigos, 380304.85E/8611305.81, a.s.l.: 290 m., 26.vi–3.vii.2008 (I. Gómez leg.), 1♀, ZMUT; idem, but 7–14.viii.2008, 1♀, ZMUT; idem, but 14–21.viii.2008, 2♀♀, ZMUT; idem, but Tambopata, Explorer’s inn, 12°50'30"S, 69°17'31"W, 161 m., 15.vi.2009, Colecta manual (M. Alvarado leg.), 1♀, ZMUT; Cusco, La Convención, Echarate, CC Timpia, 12°06'47,04"S, 72°49'34,56"W, 519 m., Bosque húmedo de montana, 29.i.2010, Light (C. Espinoza & E. Rázuri leg.), 1♀, ZMUT; idem, but Reserva Comunal Amarakaeri, 12°55'S, 70°51'W, 333–884 m., 17.ix–14.xi.2010, Malaise trap (M. Vilchez & C. Castillo leg.), 1♀, ZMUT; Loreto, Qda. Pucacuro, 18M 0501611E/9726184N, Bosque de terraza media, 173 m., 24.x.2008, Malaise trap. (M. Vilchez leg.), 1♀, ZMUT; idem, but Pucallpa, 2011 (I. Gómez leg.), 1♀, ZMUT; idem, but Iquitos area, Allpahuayo, 30°58'00"S, 73°25'16"W, 24–30.x.2011 (Gómez & Sääksjärvi leg.), 1♀, ZMUT; idem, but 17–23.x.2011, 1♀, ZMUT; idem, but 26.ix–2.x.2011, 1♀, ZMUT; idem, but 4–10.vii.2011, 1♀, ZMUT; idem, but 14–20.xi.2011, 1♀, ZMUT; idem, but iv.2011, 1♀, ZMUT; idem, but 31.x–6.xi.2011, 1♀, ZMUT; idem, but 28.xi–4.xii.2011, 1♀, ZMUT; idem, but 25–31.vii.2011, 2♀♀, ZMUT; idem, but 22–28.viii.2011, 2♀♀, ZMUT; idem, but 7–13.xi.2011, 2♀♀, ZMUT; idem, but white sand, 24.iii–16.iv.2000 (Sääksjärvi et al. leg.), APHI, E2/4, 1♀, ZMUT; idem, but 1.xii–15.xii.2000, E1(17), 1♀, ZMUT; idem, but J1, 1.xii.2000, 1♀, ZMUT.

**Figures 107–112.**
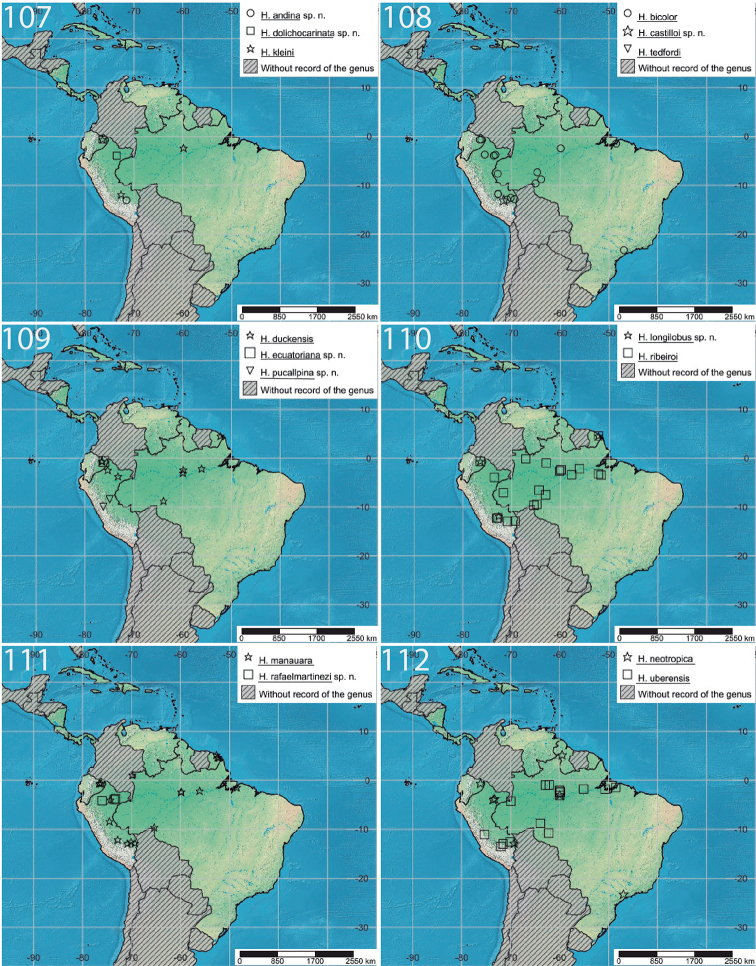
Geographic distribution of the *Hymenoepimecis* species in this study.

### Hymenoepimecis neotropica

Taxon classificationAnimaliaHymenopteraIchneumonidae

(Brues & Richardson, 1913)

A4429920-9BEB-59E5-A452-67A9A2BE0DEE

Figures 10 , 25 , 40 , 55 , 70 , 85

Epimecis neotropica Brues & Richardson, 1913: 495.
Hymenoepimecis neotropica (Brues & Richardson): Viereck, 1912: 149

#### Diagnosis.

See Pádua et al. (2015).

#### Male.

Unknown.

#### Distribution.

Brazil, Guiana, Ecuador** and Peru* (Fig. 112).

#### Biological notes.

Parasitoid of *Araneus omnicolor* (Keyserling, 1893) (Araneae: Araneidae) (Sobczak et al. 2012).

#### Material examined.

Ecuador, Dept. Orellana, Tiputini, 00°37'55"S, 76°08'39"W, a.s.l.: 220–250 m., 29.vi.1998, Fogging, Lot #1801 (T.L. Erwin et al. leg.), 1♀, ZMUT. Peru: Loreto, Maynas, Bosque ribereño, 18M 533166E/9583208N, 129 m., 23.vii.2008, Manual (C. Castillo leg.), 1♀, ZMUT; idem, but 18M 596425E/9520800N, 102 m., Pantano arbóreo, 8.vii.2008, 1♀, ZMUT; idem, but Pantano herbáceo, 18M 598912E/9522935N, 117 m., 12.vii.2008, Manual, 1♀, ZMUT; idem, but Alto Nanay, Qda. Lobilios, 119 m., Bosque de arena blanca, 18M 0565793E/9610621N, 20.xii.2008, Colecta manual, 1♀, ZMUT; idem, but 10.xii.2008, Bosque de arena blanca, 1♀, ZMUT; idem, but Albarenga north, 195 m., Colinas fuertmnt. Disect. [sic], 18M 0530961E/9646100N, 20.xi.2008, 1♀, ZMUT; idem, but 130 m., 18M 0532028E/9647431N, Bosque de Terraza media, 11.xi.2008, 1♀, ZMUT; idem, but Iquitos area, Allpahuayo, 22.v–11.vi.2000, Malaise trap, white sand (I.E. Sääksjärvi et al. leg.), APHI, E3/7, 1♀, ZMUT; idem, but 16.vii–02.viii.2000, clay, APHI, J1/10, 1♀, ZMUT; idem, but 04–17.x.2000, APHI, H1/14, 1♀, ZMUT; idem, but 30°58'00"S, 73°25'16"W, 12–18.ix.2011 (Gómez & Sääksjärvi leg.), 1♀, ZMUT; idem, but Pucallpa, 7.vi.1963 (J.M. Schunke leg.), 1♀, ZMUT; idem, but Rio Nanay, ca. Diamante azul, Colinas fuertmnt disect [sic], 131 m., 18M 638497E/9570148N, 10.xi.2008, Manual (L. Huerto leg.), 1♀, ZMUT idem, but Dept. of Loreto, Iquitos area, Allpahuayo, Malaise trap, (I.E. Sääksjärvi et al. leg.), 11–16.viii.2000, 1♀, ZMUT; idem, but J1, 20.i.2001, 1♀, ZMUT; idem, but F1(3), 8.iii.2000, 1♀, ZMUT; idem, but I1 (11), 18.vii.2000, 1♀, ZMUT; Madre de Dios, Tambopata, NNRR, Explorer’s inn, 12°50'S, 69°17'W, 189 msnm, 15–18.v.2008 (L. Figueroa & M. Alvarado leg.), 1♀, ZMUT; idem, but 18–19.xii.2008, Malaise trap (M. Alvarado & L. Sulca leg.), 1♀, ZMUT.

### Hymenoepimecis pucallpina

Taxon classificationAnimaliaHymenopteraIchneumonidae

Pádua & Sääksjärvi sp. nov.

FF6A28DE-3E79-596A-88B1-705002F9C4D0

http://zoobank.org/67D0DB27-DACB-42CB-8634-F4BD43FDB95A

Figures 11 , 26 , 41 , 56 , 71 , 86

#### Diagnosis.

This species can be distinguished from all other *Hymenoepimecis* by the combination of the following characters: 1) fore wing black, with a yellowish band between junction of vein R1 up to pterostigma until half vein M; 2) metasoma entirely black; 3) hind leg black; 4) occipital carina projected and curved upwards dorsally.

#### Description.

**Female.** Body approx. [16.0] mm; face [1.0] times as broad as high, smooth, slightly convex with few spaced bristles; head in dorsal view, with gena slightly narrowed behind eyes; posterior ocelli separated from eyes by approx. [1.2] times its own maximum diameter; occipital carina projected and curved upwards dorsally. Pronotum long, smooth and polished, with distance from tegula to head greater than [0.6] times distance from tegula to hind margin of propodeum, and in anterior part with opening pocket-like structure not reduced longitudinally; mesoscutum smooth and polished; scutellum, in profile, convex; mesopleuron smooth and polished, with anterodorsal and posterodorsal parts bearing sparse, fine setiferous punctures; metapleuron smooth and polished, with a few sparse, fine setiferous punctures; propodeum smooth, polished, with sparse, fine setiferous punctures and with lateral longitudinal carina present only posteriorly. Fore wing approx. [13.0] mm; cu-a interstitial to the base of Rs&M; 2rs-m approx. [0.75] times as long as abscissa of M between 2rs-m and 2m-cu; hind wing with abscissa of Cu1 meet cu-a equidistant between 1A and M. Hind leg with tibia + tarsus [0.6] times the fore wing length; tarsal claw with more or less square basal lobe with apex of claw overtaking the lobe. Metasoma slender; tergite I [1.6] times as long as posteriorly width, centrally quite strongly convex with lateral carinae present only at extreme anterior end flanking the anterior concavity; sternite I with a low, rounded swelling posteriorly; tergite II approx. [1.3] times as long as posteriorly width; tergites III–IV approx. [1.2] times as long as posteriorly width; ovipositor [1.6] times as long as hind tibia.

***Colour.*** Head black with apical margin of clypeus and mouthparts (except apex mandible black) yellowish; antenna blackish. Mesosoma entirely orange. Fore and mid leg orange, the hind leg black. Fore wing black, with basal region yellowish and with a yellowish band between junction of vein R1 up to pterostigma until half vein M; pterostigma black, except apical margin yellowish; hind wing black with basal region and apex yellowish. Metasoma entirely black; ovipositor brownish with apex reddish brown, sheath blackish.

#### Male.

Unknown.

#### Distribution.

Peru (Fig. 109).

#### Biological notes.

Host unknown.

#### Etymology.

The specific name refers to the name given to people born in the city of Pucallpa, Peru.

#### Type material.

***Holotype*** ♀. Peru, Dept. Loreto, Pucallpa, 15.iv.1950 (J.M. Schunke leg.), B.M. 1950-559 [sic], NHM. ***Paratypes***: Dept. Huánuco, Tingo Maria, Cueva de Las Pavas, 23–27.vii.1982 (C. Porter & T. O’neill leg.), 6♀♀, FSCA; idem, but 12–15.vii.1974 (C. Porter & L. Stange leg.), 2♀♀, FSCA; idem, but 20–27.i.1968 (A. Garcia & C. Porter leg.), 1♀, FSCA.

#### Comments.

*Hymenoepimecis pucallpina* sp. nov. closely resembles *H. uberensis* Pádua & Onody, 2015 and *H. ribeiroi* Pádua & Oliveira, 2015 mainly by having fore wing black with a yellowish band between junctions of vein R1 up to pterostigma until half vein M. It differs from both of these species by having the metasoma entirely black and occipital carina projected, and curved upwards dorsally (occipital carina not curved upwards and with a concavity in the apex dorsally in *H. uberensis*, occipital carina with dorsal margin reduced, in profile view in *H. ribeiroi* and metasoma orange, with tergites VI+ black in both species).

### Hymenoepimecis rafaelmartinezi

Taxon classificationAnimaliaHymenopteraIchneumonidae

Pádua & Sääksjärvi sp. nov.

BAFFA4D7-1D45-5A39-8FB2-E6BE550A635C

http://zoobank.org/59F648CD-2ED2-4E2E-9A0E-B7B609995608

Figures 12 , 27 , 42 , 57 , 72 , 87 , 104

#### Diagnosis.

This species can be distinguished from all other *Hymenoepimecis* by the combination of the following characters: 1) fore wing hyaline yellowish, with two blackish bands; 2) face sculptured below the insertion of antennae, with a longitudinal carina in the middle part; 3) occipital carina projected, not curved upwards, with a concavity in the apex dorsally; 4) pronotum with the pocket-like structure reduced longitudinally; 5) sternite I with a ventral spine-like projection posteriorly; 6) metasoma orange, with tergites VI+ black; 7) hind leg orange, with femur, tibia and tarsus back; 8) female with tarsal claw with basal lobe more or less square and apex of claw overtaking the lobe; 9) female with ovipositor 1.0–1.2 times as long as hind tibia.

#### Description.

**Female.** Body approx. [10.0] 9.0–10.0 mm; face [0.9] 0.8–1.1 times as broad as high, sculptured below the insertion of antennas, with longitudinal carina in the middle part and with few bristles spaced on the lower face; head in dorsal view with gena strongly narrowed behind eyes; posterior ocelli separated from eyes by [1.0] 0.8–1.0 times its own maximum diameter; occipital carina not curved upwards, with a concavity in the apex dorsally. Pronotum long, smooth and polished, with distance from tegula to head greater than [0.5] 0.5–0.6 times distance from tegula to hind margin of propodeum, and in anterior part with opening pocket-like structure reduced longitudinally; mesoscutum smooth and polished; scutellum, in profile, convex; mesopleuron smooth and polished, with anterodorsal and posterodorsal parts bearing sparse, fine setiferous punctures; metapleuron smooth and polished, rather uniformly covered with sparse, fine setiferous punctures; propodeum smooth, polished, with sparse, fine setiferous punctures and with lateral longitudinal carina present only posteriorly. Fore wing approx. [7.0] 7.0–8.0 mm; cu-a interstitial to the base of Rs&M; 2rs-m [0.3] 0.3–0.5 times as long as abscissa of M between 2rs-m and 2m-cu; abscissa of Cu1 meeting 1m-cu equidistant between Cu1a and Cu1b; hind wing with abscissa of Cu1 meeting cu-a closer to 1A that M. Hind leg with tibia + tarsus [0.55] 0.5–0.6 times the fore wing length; tarsal claw with basal lobe slightly more or less square, with apex of claw overtaking the lobe. Metasoma slender; tergite I approx. [1.2] 1.2–1.4 times as long as posteriorly width, centrally quite strongly convex with lateral carinae present only at extreme anterior end flanking the anterior concavity; sternite I with a ventral projection, spine-like, posteriorly; tergite II [1.1] 1.0–1.1 times as long as posteriorly width; tergites III–IV [1.0] 1.0–1.05 times as long as posteriorly width; ovipositor [1.15] 1.0–1.2 times as long as hind tibia.

***Colour.*** Head black; clypeus with apical margin yellowish, labrum and mouthparts yellowish, except apex mandible black; antenna brown. Mesosoma orange. Fore and mid leg orange, the hind leg orange, with femur, tibia and tarsus black. Fore wing hyaline yellowish, fore wing hyaline yellowish, with apex blackish and with a blackish preapical band; pterostigma blackish brown; hind wing blackish with base and apex slightly yellowish. Metasoma orange, with tergite V with posterior margin black and tergites VI+ black; ovipositor brownish and sheath blackish brown.

#### Male.

(Fig. 104). Similar to female in structure and colouration, but with body with 7.0–8.5 mm; face 0.9–1.0 times as broad as high; posterior ocelli separated from eyes by 0.8–0.9 times its own maximum diameter. Fore wing 6.0–7.0 mm; cu-a more or less interstitial to the base Rs&M; 2rs-m 0.3–0.4 times as long as abscissa of M between 2rs-m and 2m-cu. Tarsal claw tarsal claw simple. Metasoma slender; tergite I 1.3–1.6 times as long as posteriorly width; tergite II 1.0–1.1 times as long as posteriorly width; tergites III–IV 0.95–1.1 times as long as posteriorly width.

#### Distribution.

Peru (Fig. 111).

#### Biological notes.

Host unknown.

#### Etymology.

The specific name refers to a young boy called Rafael Martinez, a friend of Yves Braet. Yves Braet has sent us many Darwin wasp samples from French Guiana, and Rafael has helped Yves in science.

#### Type material.

***Holotype*** ♀. Peru, Loreto, Maynas, Qda. Aguablanca, 151 m., Terrazas medias, 18M 0521603E/9676612N, 24.i.2009, Malaise trap (F. Meza leg.), ZMUT. ***Paratypes***: idem holotype, but 18M 534463E/9584008N, Bosque rebereño, 115 m., 23.vii. 2008, Manual (C. Castillo leg.), 1♂ and 1♀, ZMUT; idem, but Rio Itaya, Colinas fuertmnt. disect., 18M 0635650E/9528654N, 127 m., 11.ii.2009, Malaise trap (W. Paredes leg.), 1♂, ZMUT; idem, but Rio Copalyacu, 03°42'59"S, 75°26'00"W, 165 m., 8.xii.2009, Malaise trap (L. Sulca leg.), 1♂, ZMUT; idem, but Rio Urituyacu, ca. Ayahuasca, 04°09'46"S, 76°00'49"W, 146 m., 19.xi.2009, Manual, 1♀, ZMUT; idem, but Mrg. Izq. Rio Nanay, Colinas fuertmnt. disect., 18M 0574494E/9614852N, 163 m., 23.i.2009, Flight-intercept trap (W. Paredes leg.), 1♀, ZMUT; idem, but Iquitos area, Allpahuayo, 2–18.viii.2000, clay, Malaise trap (Sääksjärvi et al. leg.), APHI, H1/11, 1♂, ZMUT; idem, but 19.ix–4.x.2000, APHI, J2/13, 1♂, ZMUT; idem, but 20.ii–8.iii.2000, APHI, H2/2, 1♂, ZMUT; 17.x–8.xi.2000, APHI, H2/15, 1♂, ZMUT; idem, but APHI, H1/15, 2♂♂, ZMUT; idem, but 3–22.v.2000, APHI, H1/6, 1♂, ZMUT; idem, but 18.viii–14.ix.2000, APHI, G2/12, 1♂, ZMUT; idem, but APHI, H2/12, 2♂♂ and 1♀, ZMUT; idem, but 8.iii–24.iii.2000, APHI, H1/3, 1♂, ZMUT; idem, but 22.v–11.vi.2000, APHI, H1/7, 1♂, ZMUT; idem, but 17.iv–3.v.2000, APHI, H1/5, 2♂♂, ZMUT; idem, but 2–24.iii.2000, APHI, G1/3, 1♂, ZMUT; idem, but 30°57'84"S, 73°25'39"W, 5–11.xii.2011 (Gómez & Sääksjärvi et al. leg.), 1♂, ZMUT; idem, but 7–13.xi.2011, 1♂, ZMUT; Dept. of Loreto, Iquitos area, Allpahuayo, Malaise trap, (I.E. Sääksjärvi et al. leg.), H1, 23.i.2001, 1♂, ZMUT; idem, but H2(12), 14.ix.2000, 1♂, ZMUT; idem, but I1(16), 15.xii.2000, 1♂, ZMUT; idem, but G1(15), 8.xi.2000, 1♂, ZMUT; idem, but H1, 1.xii.2000, 76, 1♂, ZMUT; idem, but H1, 23.i.2001, 1♂, ZMUT; idem, but G2(7), 11.vii.2000, 97, 1♂, ZMUT; idem, but I1, 1.xii.2000, 42, 1♀, ZMUT; idem, but J1, 1.xii.2000, 16, 1♀, ZMUT.

#### Comments.

*Hymenoepimecis rafaelmartinezi* sp. nov. closely resembles *H. uberensis* Pádua & Onody, 2015, mainly by having wings bicoloured, face sculptured below the insertion of antennas, with longitudinal carina in the middle part and with a few bristles spaced on the lower face, and by sternite I with a ventral spine-like projection posteriorly. It differs from *H. uberensis* mainly by having the fore wing hyaline yellowish with two black bands and hind leg orange, with femur, tibia and tarsus black (fore wing blackish, with yellowish hyaline band between junction of vein R1 up to pterostigma until half vein M and hind leg entirely black in *H. uberensis*).

### Hymenoepimecis ribeiroi

Taxon classificationAnimaliaHymenopteraIchneumonidae

Pádua & Sobczak, 2015

46EE9ACB-6316-5FDB-864F-413D197A4679

Figures 13 , 28 , 43 , 58 , 73 , 88 , 105

Hymenoepimecis ribeiroi Pádua & Sobczak, 2015: 188.

#### Diagnosis.

See Pádua et al. (2015).

#### Distribution.

Brazil, French Guiana* and Peru* (Fig. 110).

#### Biological notes.

Host unknown.

#### Material examined.

French Guiana, M. de Kaw, Patawa (PM), ii.2003 (O. Morvan leg.), 1♀, ZMUT; Saül, 13.xii.2011, Malaise trap (without collector), 1♀ and 1♂, ZMUT. Ecuador: Dept. Orellana, Onkonegare, 00°39'25,7"S, 76°27'10,8"W, a.s.l.: 216.3 m., 9.ii.1995, Fogging, Lot #985 (T.L. Erwin et al. leg.), 1♂, ZMUT. Peru, Dept. of Loreto, Iquitos area, Allpahuayo, 17.xi–3.xii.1998, varillal, Malaise trap (I.E. Sääksjärvi, R. Jussila et al. leg.), APHI D2/7, 2♂♂, ZMUT; idem, but 17.xii–20.i.1998, APHI D1/9, 1♂, ZMUT; idem, but APHI C2/9, 1♂ and 1♀, ZMUT; idem, but 18.ix–4.x.1998, clay, APHI C2/3, 1♂, ZMUT; idem, but 4–20.x.1998, APHI C2/4, 2♂♂, ZMUT; idem, but 7–17.x.2000 (Sääksjärvi et al. leg.), APHI, H1/14, 1♂, ZMUT; idem, but 11–29.vi.2000, 1♂, ZMUT; idem, but 17.iv–3.v.2000, APHI, H1/5, 2♂♂, ZMUT; idem, but 16.vii–2.viii.2000, APHI, H1/10, 2♂♂, ZMUT; idem, but APHI, H2/10, 1♀, ZMUT; idem, but APHI, I1/10, 1♂, ZMUT; idem, but APHI, G1/10, 1♂, ZMUT; idem, but 18.viii–14.ix.2000, APHI, H2/12, 2♂♂ [one without head], ZMUT; idem but APHI, I1/12, 1♂, ZMUT; idem, but APHI, E3/12, 1♀, ZMUT; idem, but APHI, J2/12, 1♀, ZMUT; idem, but 20.ii–2.iii.2000, white sand, APHI, G1/2, 1♂, ZMUT; idem but 14.ix–4.x.2000, 1♂, ZMUT; idem, but 19.ix–4.x.2000, APHI, I1/13, 2♂♂, ZMUT; idem, but 15.x–8.xi.2000, APHI, I1/15, 2♂♂, ZMUT; idem, but 4–15.x.2000, APHI I1/14, 2♂♂, ZMUT; idem, but 17.x–8.i.2000, APHI, G2/15, 1♂, ZMUT; idem, but 30°57'84"S, 73°25'39"W, 5–11.xii.2011 (Gómez & Sääksjärvi leg.), 1♂, ZMUT; idem, but 14–20.xi.2011, 1♂, ZMUT; idem, but Mishana, clayish soil, 16.x–1.xi.1998 (I.E. Sääksjärvi, R. Jussila et al. leg.), APHI, A1/5, 1♂, ZMUT; idem, but 1–16.xii.1998, clay, APHI, A1/8, 1♂, ZMUT; idem, but Maynas, Bosque de Terraza media, 18M 500248E/9624121N, 143 m., 2.viii.2008, Malaise trap (C. Castillo leg.), 1♂, ZMUT; idem but Alto Nanay, Albarenga north, 128 m., 18M 0533061E/9645180N, Terrazas bajas inund., 24.xi.2008, Flight intercept trap (C. Castillo leg.), 1♂ [without metasoma], ZMUT; idem, but 157 m., 0532439E/9646162N, Colinas bajas fuertmnt. dissect., 17.xi.2008, Colecta manual, 1♂, ZMUT; idem, but 130 m., 0532028E/9647431N, Bosque de terraza media, 29.xi.2008, Malaise trap, 2♂, ZMUT; idem, but 0532028E/9647431N, 28.xi.2008, Colecta manual, 1♀, ZMUT; idem, but Qda. Lobillos, 119 m., 0565793E/9610621N, Colinas fuertmnt. dissect., 19.xii.2008, 1♀, ZMUT; Dpto. Madre de Dios, Los Amigos, 382633,452E/8610288,894N, a.s.l.: 241.7 m., 17–24.vii.2008, Malaise trap (I. Gómez leg.), 1♀, ZMUT; idem, but Explorer’s Inn Amazon logde, 161 m., 12°50'30"S, 69°17'31"W, 13.ix.2009, Malaise trap (L. Sulca leg.), 1♂, ZMUT; Cusco, La Convención, 12°19'21,26"S, 73°02'44,08"W, 792 m., Bosque premontano, 26.iv.2007, Malaise trap (W. Paredes leg.), 2♂♂, ZMUT; idem, but Echarate, CC Kitaparay, 12°12'51,79"S, 72°50'04,31"W, 608 m., 8–11.xi.2009 (C. Espinoza & E. Razuri leg.), 1♂, ZMUT; idem, but Reserva Comunal Amarakaeri, 12°55'S, 70°51'W, 333–884 m., 17.ix–14.xi.2010, Malaise trap (M. Vilchez & C. Castillo leg.), 2♂♂, ZMUT; Dept. of Loreto, Iquitos area, Allpahuayo, Malaise trap, (I.E. Sääksjärvi et al. leg.), H1(16), 21.xii.2000, 1♂, ZMUT; idem, but I1, 1.xii.2000, 1♂, ZMUT; idem, but I1(16), 15.xii.2000, 1♂, ZMUT; idem, but H1(16), 21.xii.2000, 1♂, ZMUT; idem, but I1, 1.xii.2000, 1♂, ZMUT; idem, but I1, 1.xii.2000, 1♂, ZMUT; idem, but I1, 20.i.2001, 1♂, ZMUT; idem, but H1(4), iv.2000, 1♂, ZMUT; idem, but I1(16), 15.xii.2000, 1♂, ZMUT.

### Hymenoepimecis tedfordi

Taxon classificationAnimaliaHymenopteraIchneumonidae

Gauld, 1991

8AD18BF8-9D16-5531-A90C-5069E117E464

Figures 14 , 29 , 44 , 59 , 74 , 89

Hymenoepimecis tedfordi Gauld, 1991: 340.

#### Diagnosis.

This species can be distinguished from all other *Hymenoepimecis* by the combination of the following characters: 1) fore wing hyaline; 2) mesosoma orange with propleuron, pronotum, metapleuron ventrally, and propodeum black (metapleuron entirely orange in Nicaraguan specimens); 3) epicnemial carina present ventrally, sometimes visible laterally; 4) metasoma entirely blackish; 5) female with ovipositor 1.0–1.1 times as long as hind tibia.

#### Distribution.

Costa Rica and Nicaragua* (Fig. 108).

#### Biological notes.

Parasitoid of *Leucauge mariana* (Keyserling, 1881) (Araneae: Tetragnathidae) (Gauld, 1991; Eberhard, 2013).

#### Material examined.

Nicaragua, Jinotega, RN Cerro Kilambé, 1310 ± 10 m., 13.56541/-85.69785, Pasture/cloud, forest edge, 22–26.v.2011, Malaise trap (without collector), LLAMA#Ma-D-05-1-01 [sic], 1♀, ZMUT.

### Hymenoepimecis uberensis

Taxon classificationAnimaliaHymenopteraIchneumonidae

Pádua & Onody, 2015

73392335-2261-5C02-BEA5-76C6654C4FB3

Figures 15 , 30 , 45 , 60 , 75 , 90 , 106

Hymenoepimecis uberensis Pádua & Onody, 2015: 190.

#### Diagnosis.

See Pádua et al. (2015).

#### Distribution.

Brazil and Peru* (Fig. 112).

#### Biological notes.

Host unknown.

#### Material examined.

Peru, La Merced, Fundo Genova, 21.vi.2008, Malaise trap (without collector), AECID: A/013484/07, 1♀, ZMUT; idem, but 7.vi.2008, 1♂, ZMUT; Cusco, ca. P.V. Tono, 12°57'48"S, 71°32'06"W, 862 m., 26.ix.2007, Malaise trap (C. Castillo leg.), 1♂, ZMUT; Dept. Madre de Dios, Los Amigos, 380955, 769E/8610042,474N, a.s.l.: 240.2 m., 26.vi–3.vii.2008, Malaise trap (I. Gómez leg.), 1♀, ZMUT; idem, but 380792,164E/8610919,14N, a.s.l.: 280.5 m., 7–14.viii.2008, 1♂, ZMUT; Marcapata [without others information], NHRS-HEVA #2908, 1♀, ZMUT.

## Discussion

The present study increases the number of recognized *Hymenoepimecis* species to 27 species. In addition, is presented a large amount of faunistic records from different parts of Central and South America. It is also shown that *H. rafaelmartinezi* sp. nov. belongs to the *H. jordanensis* species group (Pádua et al. 2015) which is characterised by the following set of characters: 1) face sculptured below the insertion of antennae, with a longitudinal carina in the middle part of the face; 2) head with occipital carina projected, not curved upwards, with a dorsal concavity in the apex; 3) pronotum with the pocket-like structure reduced longitudinally; 4) sternite I with a ventral spine-like projection posteriorly. After the present study this species group is composed of five species (*H. amazonensis*, *H. jordanensis*, *H. kleini*, *H. rafaelmartinezi* sp. nov., and *H. uberensis*),

A noteworthy new character state was recorded for *Hymenoepimecis*: in *H. dolichocarinata* sp. nov., the epicnemial carina is ventrally present and extending to the level of the lower lateral corner of the pronotum. This character state is shared with most species of *Acrotaphus* (Pádua et al. 2020), and likely represents a homoplastic trait in the two genera.

Finally, the genus is reported for the first time from the Andes (Figs 107, 108). Two Peruvian species, both collected by Carol Castillo, were found from the Andean-Amazonian interface (Cusco, San Pedro, Cosñipata valley, 1302–1520 m). *Hymenoepimecis andina* sp. nov. and *H. castilloi* sp. nov. are both remarkable because they resemble *Polysphincta* Gravenhorst (and *Ticapimpla amazonica* Palacio, Broad, Sääksjärvi & Veijalainen, 2010) by the shape of the occiput. All other species of *Hymenoepimecis* have the occipital carina strongly raised and produced into a neck-like structure.

## Supplementary Material

XML Treatment for Hymenoepimecis

XML Treatment for Hymenoepimecis andina

XML Treatment for Hymenoepimecis bicolor

XML Treatment for Hymenoepimecis castilloi

XML Treatment for Hymenoepimecis dolichocarinata

XML Treatment for Hymenoepimecis duckensis

XML Treatment for Hymenoepimecis ecuatoriana

XML Treatment for Hymenoepimecis kleini

XML Treatment for Hymenoepimecis longilobus

XML Treatment for Hymenoepimecis manauara

XML Treatment for Hymenoepimecis neotropica

XML Treatment for Hymenoepimecis pucallpina

XML Treatment for Hymenoepimecis rafaelmartinezi

XML Treatment for Hymenoepimecis ribeiroi

XML Treatment for Hymenoepimecis tedfordi

XML Treatment for Hymenoepimecis uberensis

## References

[B1] Brues CT Richardson CH (1913) Descriptions of new parasitic Hymenoptera from British Guiana. Bulletin of the American Museum of Natural History 32: 485–503.

[B2] Brullé MA (1846) Tome Quatriene. Des Hymenopteres. Les Ichneumonides. Lepeletier de Saint-Fargeau A. “Histoire Naturelles des Insectes.” Paris, 680 pp.

[B3] Cresson ET (1865) On the Hymenoptera of Cuba. Proceedings of the Entomological Society of Philadelphia 4: 1–200.

[B4] Eberhard WG (2013) The Polysphinctine Wasps *Acrotaphus tibialis*, *Eruga* ca. *gutfreundi*, and *Hymenoepimecis tedfordi* (Hymenoptera, Ichneumonidae, Pimplinae) Induce Their Host Spiders to Build Modified Webs. Annals of the Entomological Society of America 106(5): 652–660. 10.1603/AN12147

[B5] Gauld ID (1991) The Ichneumonidae of Costa Rica I. Memoirs of the American Entomological Institute 47: 1–589.

[B6] Gauld ID (2000) The re-definition of Pimplinae genus *Hymenoepimecis* (Hymenoptera: Ichneumonidae) with a description of a plesiomorphic new Costa Rica species. Journal of Hymenoptera Research 9: 213–219.

[B7] Gonzaga MO Sobczak JF Penteado-Dias AM Eberhard WG (2010) Modification of *Nephila clavipes* (Araneae, Nephilidae) webs induced by the parasitoids *Hymenoepimecis bicolor* and *H. robertsae* (Hymenoptera, Ichneumonidae). Ethology Ecology & Evolution 22: 151–165. 10.1080/03949371003707836

[B8] Kriechbaumer J (1890) Ichneumoniden-Studien. Neue Ichneumoniden des Wiener Museums. II. Annalen des Naturhistorischen Hofmuseums Wien 5: 479–491.

[B9] Kumagai AF Graf V (2002) Biodiversidade de Ichneumonidae (Hymenoptera) e monitoramento das espécies de Pimplinae e Poemeniinae do Capão da Imbuia, Curitiba, Párana. Revista Brasileira de Zoologia 19(2): 445–452. 10.1590/S0101-81752002000200010

[B10] Loffredo APS Penteado-Dias AM (2009) New species of *Hymenoepimecis* Viereck (Hymenoptera, Ichneumonidae, Pimplinae) from Brazilian Atlantic forest. Revista Brazileira de Entomologia 53: 11–14. 10.1590/S0085-56262009000100004

[B11] Pádua DG Sääksjärvi IE Monteiro RF Oliveira ML (2020) Review of the New World genus *Acrotaphus* Townes, 1960 (Hymenoptera: Ichneumonidae: Pimplinae), with descriptions of fifteen new species. Zootaxa 4719: 1–062. 10.11646/zootaxa.4719.1.1 32230647

[B12] Pádua DG Oliveira ML Onody HC Sobczak JF Sääksjärvi IE Gómez IC (2015) The Brazilian Amazonian species of *Hymenoepimecis* Viereck, 1912 (Hymenoptera: Ichneumonidae: Pimplinae). Zootaxa 4058(2): 175–194. hhttps://doi.org/10.11646/zootaxa.4058.2.2 26701518

[B13] Pádua DG Salvatierra L Sobczak JF Oliveira ML (2016) Parasitism of *Hymenoepimecis manauara* Pádua & Oliveira (Hymenoptera: Ichneumonidae: Pimplinae) on *Leucauge henryi* Mello-Leitão (Araneae: Tetragnathidae) in Brazilian Amazonian. Biodiversity Data Journal 4: e11219. 10.3897/BDJ.4.e11219 PMC5267549 28174511

[B14] Sobczak JF Loffredo APS Penteado-Dias AM Gonzaga MO (2009) Two new species of *Hymenoepimecis* (Hymenoptera: Ichneumonidae: Pimplinae) with note on their spider hosts and behavior manipulation. Journal of Natural History 43(43): 2691–2699. 10.1080/00222930903244010

[B15] Sobczak JF Loffredo APS Camargo LF Penteado-Dias AM (2012) *Hymenoepimecis neotropica* (Brues & Richardson) (Hymenoptera, Ichneumonidae, Pimplinae) parasitoid de *Araneus omnicolor* (Keyserling) (Araneae, Araneidae): first host record and new occurrence to Brazil. Revista Brasileira de Entomologia 56(3): 390–392. 10.1590/S0085-56262012005000055

[B16] Townes H Townes M (1966) A catalogue and reclassification of neotropic Ichneumonidae. Memories of the American Entomological Institute 8: 1–367. 10.1080/00222930903244010

[B17] Wahl DB Gauld ID (1998) The cladistics and higher classification of the Pimpliformes (Hymenoptera: Ichneumonidae). Systematic Entomology 23: 265–298. 10.1046/j.1365-3113.1998.00057.x

[B18] Yu DS van Achterberg C Horstmann K (2012) World Ichneumonoidea 2011: Taxonomy, Biology, Morphology and Distribution. Taxapad 2012, Vancouver. htpp://www.taxapad.com/ [accessed 20 May 2018]

